# Sexual selection predicts the rate and direction of colour divergence in a large avian radiation

**DOI:** 10.1038/s41467-019-09859-7

**Published:** 2019-04-16

**Authors:** Christopher R. Cooney, Zoë K. Varley, Lara O. Nouri, Christopher J. A. Moody, Michael D. Jardine, Gavin H. Thomas

**Affiliations:** 1 Department of Animal and Plant Sciences, University of Sheffield, Western Bank, Sheffield, S10 2TN UK; 2 School of Biological Sciences, Royal Holloway, University of London, Egham Hill, Egham, TW20 0EX UK; 3 Research Department of Genetics, Evolution and Environment, University College London, Gower Street, London, WC1E 6BT UK; 4 Bird Group, Department of Life Sciences, The Natural History Museum, Tring, HP23 6AP UK

## Abstract

Sexual selection is proposed to be a powerful driver of phenotypic evolution in animal systems. At macroevolutionary scales, sexual selection can theoretically drive both the rate and direction of phenotypic evolution, but this hypothesis remains contentious. Here, we find that differences in the rate and direction of plumage colour evolution are predicted by a proxy for sexual selection intensity (plumage dichromatism) in a large radiation of suboscine passerine birds (Tyrannida). We show that rates of plumage evolution are correlated between the sexes, but that sexual selection has a strong positive effect on male, but not female, interspecific divergence rates. Furthermore, we demonstrate that rapid male plumage divergence is biased towards carotenoid-based (red/yellow) colours widely assumed to represent honest sexual signals. Our results highlight the central role of sexual selection in driving avian colour divergence, and reveal the existence of convergent evolutionary responses of animal signalling traits under sexual selection.

## Abstract (web summary)

What factors explain variation in the pace and trajectory of evolutionary divergence between lineages? Here, the authors show that a proxy measure for sexual selection intensity predicts both the rate and direction of plumage colour evolution in a diverse radiation of New World passerine birds.

## Introduction

A central problem in evolutionary biology is to explain the processes shaping the evolutionary diversification of species’ phenotypic traits^1–3^. In particular, despite a long history of study^4,5^, explaining the tremendous diversity of animal sexual signalling traits has proved particularly challenging, and our understanding of the processes driving the evolution of species’ sexual signalling traits remains highly incomplete^6,7^. Sexual signalling traits mediate the complex relationship between an organism and its environment in important ways^8^. For instance, signalling traits such as colour, patterns, and sounds are under strong selection to facilitate effective communication between individuals in a given environment, but such benefits are balanced against costs such as those associated with signal production, maintenance or display, including predation^9–11^. Furthermore, animal signalling traits often play a key role in reproductive decision-making processes such as mate choice, and particularly choice for a conspecific mate^4,12^. Evolutionary divergence in signalling traits has consequently been recognised as a potentially powerful mechanism facilitating reproductive isolation before and/or after speciation among diverging populations^13,14^. Understanding the processes driving sexual signal divergence across lineages therefore has important implications for explaining multiple facets of biodiversity, including patterns of phenotypic and species diversity among groups and across geographic regions^15^.

Sexual selection has long been viewed as a potent diversifying force in animal systems^4,12^, as preferences for novel signals can theoretically drive continuous and often rapid divergence in sexually selected traits between populations^16–18^. This view is supported by empirical studies showing that young, closely related species often differ primarily in terms of male courtship traits^19–22^, implying a role for divergent sexual selection in driving the rapid evolution of (primarily male) sexual signals during or after speciation. At a macroevolutionary scale, increased intensity of sexual selection is therefore expected to drive high rates of evolution in signalling traits. A limited number of studies have attempted to test these ideas directly using a comparative approach, but so far results linking sexual selection to elevated rates of sexual signal divergence have been mixed^23,24^.

In addition to driving trait divergence and increases in evolutionary rate, sexual selection may also play a role in determining the direction of signal evolution within lineages. Ornamental traits (i.e., those under sexual selection by mate choice) such as feather colouration, signal at least three very different types of information: species identity, attractiveness, and individual quality^25^. Signals of species identity and attractiveness are expected to be arbitrary in form but differ in terms of elaborateness. This is because the information conveyed does not depend on the exact nature of the signal, only that they are distinctive or attractive^25^. Signals of quality should converge on one or a few signal types that are more effective (or have lower costs) than others^26–28^. For example, receiver sensory biases or higher production and maintenance costs may make carotenoid-based (yellow, red) pigments more efficient or honest visual signals of individual quality than other types of colour producing mechanism (e.g., melanin or structural)^29,30^. Thus if the evolution of particular signal types is promoted or constrained by selection for signals of quality, and perceptual, developmental or energetic differences between signal production mechanisms, then the direction of trait evolution under sexual selection should be to some extent predictable. The relative roles of selection for arbitrary (identity, attractiveness) versus adaptive (quality) signals on the rate and direction of ornament evolution remains unresolved^25^.

Despite these longstanding theoretical predictions, progress in resolving the role of sexual selection in signal divergence has been slow for a number of reasons. First, although previous studies have attempted to test the impact of sexual selection on signal elaboration/divergence directly^23,24,31,32^, few have explicitly assessed the importance of sexual selection for explaining variation in both the rate and direction of phenotypic evolution at broad phylogenetic scales, and whether these patterns differ between the sexes. Second, previous studies have generally been hampered either by small sample sizes or by a lack of high quality data on species’ signalling traits, limiting the potential scope and generality of the resulting inferences. Third, such studies have also typically failed to contrast the importance of sexual selection against the role of other factors, making it difficult to assess the relative importance of sexual selection in relation to alternative mechanisms (e.g., ecological selection)^15,33,34^.

Here, we use a dataset of male and female plumage colouration based on calibrated UV/Vis digital images of museum specimens and a method for summarising species-specific rates of trait evolution across a phylogenetic tree to examine the evolutionary dynamics of plumage colouration in the Tyrannida—a large, morphological diverse radiation of New World suboscine passerine birds comprising tyrant flycatchers, cotingas, manakins, and their allies. Birds offer an ideal study system in which to test the role of sexual selection in signal evolution because of the general availability of phylogenetic, phenotypic and geographic data. Furthermore, the Tyrannida in particular are especially suited to our analysis because colour patterns (and underlying colour producing mechanisms) are highly variable across species, and because species in the group occupy a wide range of ecological and environmental niches, allowing investigation of the importance of alternative hypotheses for signal evolution^35^. Plumage dichromatism represents a widely used proxy for sexual selection intensity in birds^36^, but is likely to be an imperfect measure^37,38^. We therefore investigated the relationship between dichromatism values and independent measures of sexual selection from prior work, using both proxies to test the predictions outlined above. Our results show that sexual selection (i) is associated with elevated rates of signal divergence between related species, (ii) impacts divergence between males more than females, and (iii) generates biases in the direction of signal evolution. We conclude that sexual selection plays a key role in explaining variation in both the rate and direction of plumage colour divergence in birds.

## Results

### Evolutionary rates of plumage colour divergence

**Fig. 1:**
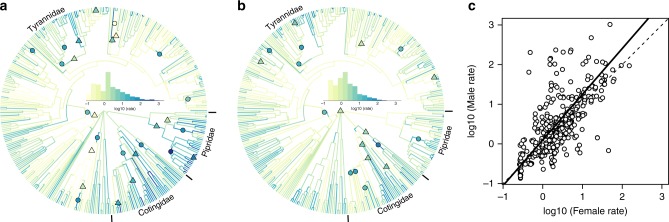
Sex-specific rate variation in plumage colour evolution across the Tyrannida phylogeny. **a**, **b** Plots showing the Tyrannida phylogeny (*n* = 372 spp.) coloured by estimates of mean relative multivariate rate of plumage colour evolution based on ten body patches for males (**a**) and females (**b**). Coloured shapes show the location of significant (posterior probability > 0.99) rate shifts affecting whole clades (triangles) and individual internal branches (circles) (shape colour reflects rate estimate; see Methods for details). Histograms (inset) show the rate distribution for each tree. **c** Relationship between (log_10_ transformed) male and female rates of plumage colour evolution (TR_ES_ values) across species. Phylogenetic reduced major axis regression (solid) and one-to-one (dashed) lines shown. Equation of the regression line: *y* = 0.10 + 1.15*x (*R*^2^ = 0.45), which is significantly different to a one-to-one slope (*T* = 3.69, d.f. = 304.08, *P* = < 0.001)

We estimated lineage-specific rates of male and female plumage colour evolution using a flexible reversible-jump model of trait evolution and mapped these onto the Tyrannida phylogeny (Fig. 1). This analysis revealed evidence for substantial heterogeneity in rates of plumage colour evolution across the Tyrannida, with rates differing by four orders of magnitude both across lineages and between the sexes (Fig. 1). In particular, we found evidence for exceptionally high rates of male plumage colour divergence (Fig. 1a) within lineages of the Pipridae (manakins; e.g., *Manacus* and *Pipra*) and Cotingidae (cotingas; e.g., *Cotinga* and *Xipholena*), as well as more isolated cases of exceptionally rapid male divergence within the Tyrannidae (tyrant-flycatchers; e.g., *Pyrocephalus rubinus*). In contrast, rates of female plumage colour divergence were found to be less extreme than in males, but nonetheless still varied by three orders of magnitude across lineages (Fig. 1b). Estimating rates of evolution for each of 10 different plumage patches (see Methods) separately revealed that patches typically associated with signalling (e.g., crown, throat, breast) have some of the fastest patch-specific rates, whereas those involved in other tasks such as locomotion (e.g., wing, tail) generally have lower rates (Supplementary Fig. 1).

Comparing species-specific trait rates (TR_ES_) of male and female plumage colour evolution estimated using a method summarising rate variation across the phylogeny (see Methods) revealed that rates of plumage colour evolution are correlated between the sexes, but that males typically diverge faster than females (Fig. 1c and Supplementary Fig. 2). The phylogenetic major axis regression line relating male and female rates was significantly greater than one, indicating that as average within-species rate increases, rates of plumage colour evolution become significantly more male biased (Fig. 1c).

### Multipredictor models of male and female plumage rates

**Fig. 2:**
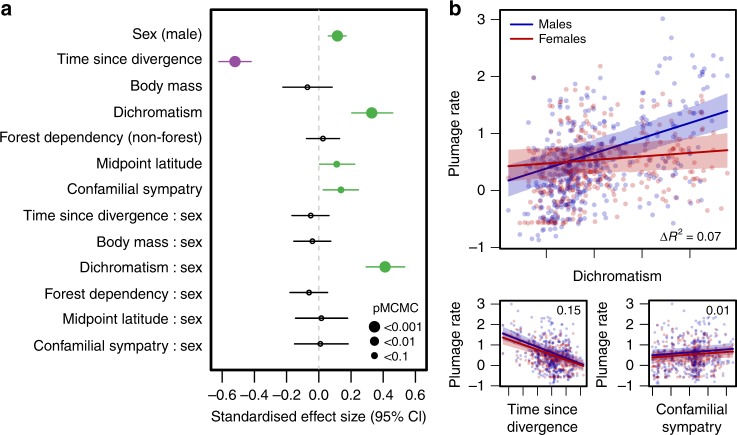
Predictors of rates of plumage colour evolution in the Tyrannida. **a** Coefficient estimates from a multipredictor BPMM predicting variation in (log_10_ transformed) whole-plumage TR_ES_ rates of colour evolution among Tyrannida species (*n* = 372). Points indicate the mean standardised effect sizes for each of the (scaled) predictor variables and lines indicate 95% credible intervals (CI). Predictors with significant (*pMCMC* *<* 0.05) effects are coloured purple (negative effect) and green (positive effect). Marginal *R*^*2*^ value (i.e., variance explained by fixed factors) for the full model is 0.23. **b** Scatterplots showing the relationship between significant predictor variables and plumage rates with regression lines (and 95% CIs) plotted for males (blue) and females (red) separately. Inset values show the change in marginal *R*^2^ (Δ*R*^2^) relative to the full model when a given predictor (and its interaction with sex) is dropped from the full model

To assess the source and strength of selection acting to drive divergence in male and female plumage colouration, we used multipredictor models of sex-specific plumage divergence rate (TR_ES_ values) to test several competing hypotheses for the evolution of plumage colouration across species. Our models revealed that a proxy for sexual selection (plumage dichromatism) has a strong positive effect on rates of male but not female plumage evolution that is independent of other key variables (Fig. 2, Supplementary Table 1). First, consistent with the results of our other analyses (Fig. 1), our models indicate that on average plumage colouration evolves more rapidly in males than females (Fig. 2). Second, our models showed that dichromatism is strongly associated with rapid rates of male plumage colour evolution, but that this effect is absent in females (Fig. 2). In other words, as the degree of dichromatism within lineages increases, rate of male plumage colour evolution tends to accelerate, whereas rates of female plumage evolution remain relatively constant (Fig. 2).

Because mean plumage dichromatism is an imperfect proxy of sexual selection we re-ran our models first accounting for varying levels of (sex-specific) intraspecific colour variation (Supplementary Table 2) and then using two alternative proxies for sexual selection. The latter models are based on: (i) maximum extent of colour differences across body regions (Supplementary Table 3) and, (ii) an independent proxy of sexual selection intensity based on scores of social mating system, sexual size dimorphism and paternal care (see Supplementary Methods) for a subset of our dataset (Supplementary Fig. 3, Supplementary Table 4). Importantly, our results are qualitatively unchanged indicating that our findings are robust to alternative approaches for indexing sexual selection intensity. Furthermore, breaking results down by patch showed largely consistent effects (Supplementary Fig. 4), except that the sex-specific effect of sexual selection (dichromatism) on colour evolution was strongest in patches assumed to be used in signalling in birds, with weak or absent effects in those patches primarily used for non-signalling functions (e.g., wing, tail).

In addition to the role of sexual selection, our multipredictor models also identified other significant effects (Fig. 2, Supplementary Table 1). First, our models revealed a consistently significant negative correlation with time since divergence, implying that young species are characterised by faster evolutionary rates relative to older species. Second, we detected marginally positive effects of midpoint latitude and confamilial sympatry. These results are consistent with the idea of latitudinal gradients in trait divergence rates and that interspecific interactions may drive trait evolution, respectively. However, these relationships were non-significant in replicate analyses using alternative datasets (Supplementary Tables 1–4). We also found no consistent effects of body mass and habitat (level of forest dependency) on rates of (male or female) plumage colour evolution across our analyses (Fig. 2, Supplementary Tables 1–4). Overall, our models explained ~18–27% of the variation in rates of male and female plumage evolution across species.

### Rapid evolution towards carotenoid-based colour signals

**Fig. 3:**
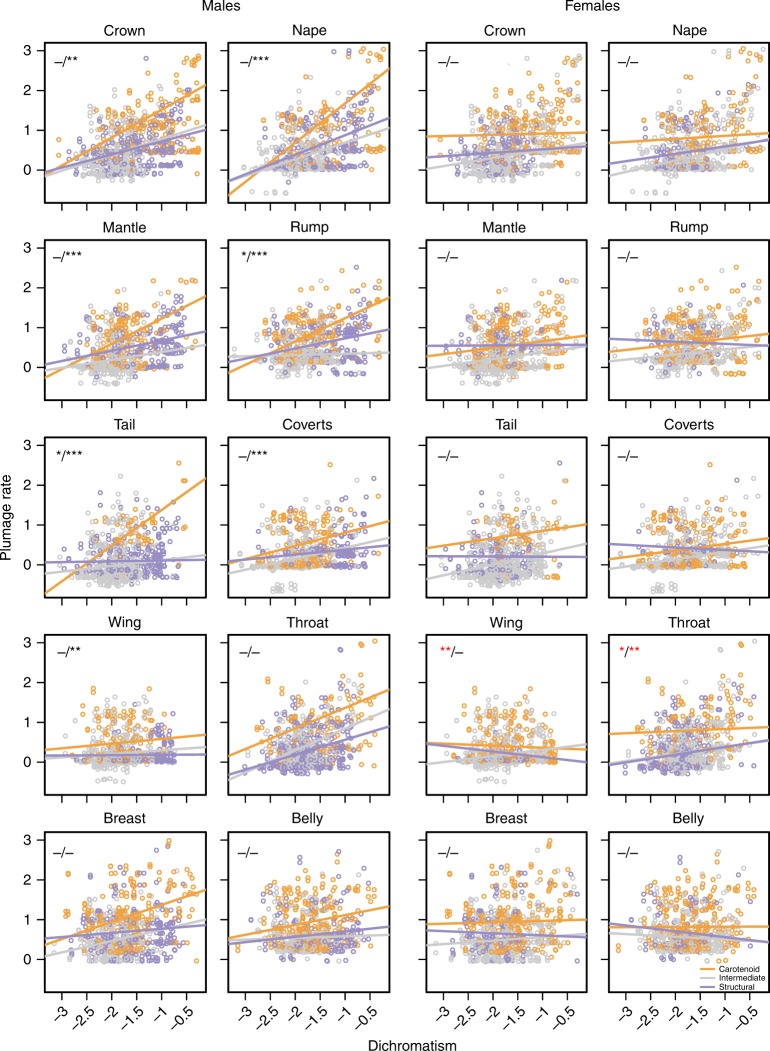
Relationships between dichromatism and rate of plumage colour evolution within body regions for different colouration types. Regression lines are plotted separately for patches consistent with carotenoid-based (orange) or structural (lilac) colouration, as well as colouration intermediate between these two extremes (grey) (see Supplementary Methods). Asterisks indicate whether the slope of the carotenoid regression line (the reference category) is significantly more positive (black) or more negative (red) compared with the slope of the (i) intermediate and (ii) structural regression lines, respectively (i.e., carotenoid slope vs. intermediate slope, carotenoid slope vs. structural slope), based on Bayesian phylogenetic mixed-models. ***pMCMC *<* 0.001; **pMCMC *<* 0.01; *pMCMC *<* 0.05; – non-significant

Our analyses thus far reveal a positive association between sexual selection (as indexed by dichromatism) and accelerated (male) plumage colour divergence across Tyrannida species. To test whether this pattern was driven by the differential response(s) of particular signal types, we re-assessed this relationship after first assigning each patch in our dataset to one of three non-overlapping categories broadly distinguishing colours consistent with two distinct colouration mechanisms (i.e., carotenoid-based and structural; see Supplementary Fig. 5 and Supplementary Methods). We then tested for an interaction between colouration category, and the slope of the relationship between dichromatism and (patch-specific) evolutionary rate. Our analysis revealed that, in males, the slope of the relationship between dichromatism and evolutionary rate is generally steeper among patches consistent with carotenoid colouration (i.e., red/orange/yellow), when compared with the other two colouration categories (Fig. 3). In other words, as the degree of dichromatism increases, carotenoid-consistent colours evolve disproportionately rapidly compared with putative structural colours, or colours intermediate between these two extremes. In contrast, in females, regression slopes for the relationship between dichromatism and evolutionary rate were generally similar among signal types (Fig. 3), with the exception of relationships across wing and throat patches, where the slope for carotenoid-consistent patches was significantly more negative (rather than more positive) than that observed for the other signal types.

**Fig. 4:**
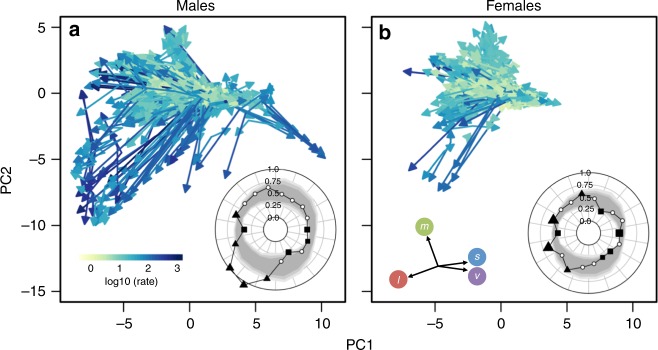
The rate and direction of plumage colour divergence among Tyrannida species. Plots combine data from 10 plumage patches for males (**a**) and females (**b**) of 372 Tyrannida species. Arrows indicate the direction of evolution and are coloured according to the corresponding patch-specific rate of evolution. Radar plots (inset, **a** and **b**) show the mean evolutionary rates of divergence trajectories (black points and lines) falling within each 20^o^ segment of two-dimensional colour space. Grey bands indicate the 95% confidence intervals for the null distribution of mean rates based on a randomisation process. Filled points indicate values showing significant (*P* < 0.05) deviations from the null distribution, with triangles and squares indicating faster and slower average rates than expected, respectively. Scale bar (inset, **a**) and vector diagram showing the loadings of receptor stimulation variables onto PC axes (inset, **b**) are relevant to both panels

We then tested whether instances of recent rapid plumage colour evolution are significantly biased towards particular evolutionary directions in colour space (Fig. 4, Supplementary Table 5). By comparing the average rate of evolution associated with different directions of recent plumage colour divergence to null expectations (see Methods), our analysis revealed that instances of fast plumage colour divergence in males are significantly biased towards the evolution of red/orange/yellow colour patches (i.e., those primarily stimulating the red cone), consistent with carotenoid-based colour producing mechanisms (Fig. 4a). In contrast, we found that evolutionary trajectories associated with the development of other colours were not significantly associated with fast rates. Specifically, evolutionary trajectories associated with the evolution of green colouration were characterised by rates of evolution that are no more rapid than expected by chance. Conversely, we found that evolution towards blue colours (i.e., those stimulating the blue/violet receptors) had on average lower rates than expected compared with a dataset-wide average. In females (Fig. 4b), consistent with our other analyses, rates of evolution in all directions were generally lower in females than in males. Nonetheless, we found some evidence that evolutionary trajectories associated with carotenoid-consistent colour patches in females were also characterised by disproportionately rapid evolutionary rates (Fig. 4, Supplementary Table 5). The results of these analyses were quantitatively similar when we ran these analyses using an alternative dataset (Supplementary Table 6).

Breaking these results down into different patches (Supplementary Fig. 6) revealed that the significant overall effects were driven primarily by significant deviations from the null expectation in only a subset of body regions typically associated with signalling and communication in birds, including the crown, nape, throat, and breast. In contrast, other body regions, in particular tail and wings, showed much weaker evidence of rate biases associated with specific directions of colour evolution. Patch-level analyses of females revealed broadly similar trends but with fewer incidences of significant bias in directions associated with elevated or suppressed rates (Supplementary Fig. 6).

## Discussion

Our results show that within the Tyrannida (tyrant flycatchers, cotingas, manakins and their allies), rates of plumage colouration are (i) correlated between the sexes, but typically faster for males than females (disproportionately so within fast-evolving species), (ii) significantly associated with proxies for sexual selection intensity (including dichromatism), with contrasting effects between the sexes, and (iii) disproportionately elevated towards carotenoid-based (i.e., red/yellow) colouration traits, particularly within body regions frequently observed to function in inter-sexual or intra-sexual displays in birds (e.g., crown, throat, breast, rump). These findings support the idea that sexual selection is the key driver in determining both the rate and direction of plumage colour divergence in birds.

We found strong support for a positive association between sexual dichromatism and the rate at which male plumage colouration traits diverge. This result is consistent with a previous study^23^ focused on a small yet broad sample of avian sister species pairs, but contrasts with a related clade-based analysis using different proxies for sexual selection intensity^24^ that found both positive and negative associations between sexual selection and ornamentation. Our results indicate that this effect holds within, as well as between avian lineages, and supports the longstanding interpretation that sexual selection accelerates the evolutionary diversification of male plumage signals involved in courtship and species recognition^4^. Furthermore, we found that on average male plumage traits diverge significantly more rapidly than female traits and that rates of female plumage divergence are not associated with dichromatism—results that are consistent with those of previous studies examining sex-specific relationships between sexual selection and plumage divergence/elaboration^23,31,32^. While overall differences in rate between the sexes may in part reflect greater constraints on plumage colouration in females than males^5^, a pattern of increasingly rapid plumage divergence rate associated with dichromatism in males (but not females) is more consistent with the general prediction that is due to sex differences in reproductive investment, sexual selection should typically be stronger on males than females^4,12^.

We find evidence for a strong between-sex correlation in plumage divergence rate that becomes disproportionately more male-biased as rate increases. One explanation for this pattern is that genetic correlations cause non-adaptive changes in female traits in response to strong divergent sexual selection acting primarily on males^39,40^. This is consistent with patterns observed for other traits, such as allometric increases in male-biased sexual size dimorphism in larger species that are driven by a correlation between evolutionary change in females and directional sexual selection on males, e.g., ref. ^41^. However, genomic studies indicate that genetic correlations between males and females may not represent a major evolutionary constraint^42–44^. An alternative explanation therefore is that correlated rates of female plumage divergence are the result of mutual selection on signalling traits in both sexes^45,46^. Evidence suggests that elaborate signalling traits are maintained in females as adaptive responses to sexual and social competition^47^. Correlated divergence rates across the sexes may therefore be the result of females co-opting (elements of) male sexual signals for use in intra-sexual or inter-sexual or social interactions. Irrespective of the underlying mechanism, however, finding that rates of plumage evolution are generally elevated in males but also correlated between the sexes suggests that sexual selection plays a role in explaining the evolutionary divergence not only of male but also female plumage traits.

Our results also reveal additional predictors of rates of plumage evolution across species. First, rates of plumage evolution were associated with time since divergence for both males and females. At face value, a negative relationship between rate and time since divergence is consistent with the hypothesis that changes in plumage colouration are associated with speciation events. However, we interpret this relationship with caution as it may alternatively be explained by a methodological bias towards inferring faster rates on shorter branches if there are errors in tree topology and/or measurement error in phenotypic data^48,49^. Second, we detected a positive but weak effect of confamilial sympatry, which is consistent with the idea that interspecific interactions may promote rather than constrain phenotypic evolution^50^. However, this relationship was absent when using an alternative tree topology and subset of the data. None of the variables included in our analysis were able to explain the significant effect of dichromatism on rates of plumage evolution and, taken together, our multipredictor modelling results imply that sexual selection is the key driver of rates of plumage colour evolution across the Tyrannida.

These conclusions are contingent on the degree to which dichromatism represents an accurate measure of variation in sexual selection intensity across lineages. Although dichromatism has been shown to correlate with independent measures of sexual selection such as social mating system and relative testes mass^31,32,51^, dichromatism represents an imperfect proxy for total sexual selection intensity. This is because other mechanisms can influence patterns of sex-differences in plumage colouration, including natural selection for female crypsis during incubation^52^ or social selection on females to signal quality in the context of male mate choice or female–female competition^47^. Furthermore, if trade-offs exist between signalling modalities, then dichromatism may underestimate sexual selection intensity, particularly in lineages emphasising acoustic over visual signalling traits^37^. However, when we tested an independent (i.e., non-dichromatism based) index of sexual selection intensity based on social mating system, sexual size dimorphism and paternal care data for a subset of our dataset, we found qualitatively identical results. Moreover, this alternative index was significantly positively correlated with our dichromatism scores (Pearson’s *r* = 0.58, *P* < 0.001), suggesting that in this system at least, dichromatism provides an adequate proxy for underlying gradients in sexual selection intensity. Nonetheless, ideally we would use a more direct measure of sexual selection intensity across lineages than any of these proxies provide, for example, indices based on variation in male mating success (e.g., Bateman gradients; ref. ^53^). However, such measures are unavailable for most species, and thus quantifiable indices such as dichromatism currently represent the most practical proxy for measuring sexual selection intensity for large numbers of species^54^. Yet, given these issues, we suggest that the effects with respect to dichromatism we report here are likely to be underestimates, implying that the true effect of sexual selection on rates of signal evolution may in fact be stronger than revealed here.

Our results also provide insight into the predictability of colour evolution under sexual selection. We found disproportionately fast rates of evolution towards areas of colour space dominated by carotenoid-based (i.e., red/yellow) colours, as well as steeper relationships between dichromatism and evolution rates for carotenoid-based patches than for other signal types (e.g., structural). The strength of these effects was strongest in front facing body regions (e.g., crown, throat, breast), consistent with a primary role for these body regions in close-proximity sexual or social signalling^55–59^. Avian carotenoid-based signals represent classic examples of sexually selected, condition-dependent signals of individual quality^29^. Although there is continuing debate regarding the precise mechanism through which carotenoid traits become honest signals^9,60^, field and behavioural studies have demonstrated the role of such traits in determining the outcome of competitive social interactions^61^. Furthermore, the genetic and biochemical basis of avian carotenoid signal production is becoming increasing well understood^62,63^, and phylogenetic evidence indicates that carotenoid pathways are deeply conserved across birds and can be readily up-regulated or down-regulated in response to relatively short-term changes in selection^64^. This flexibility contrasts with developmental and morphological constraints that potentially inhibit rapid evolution towards other types of conspicuous colouration (e.g., blue or iridescent structural colours)^65^, potentially explaining why we found that rates of evolution towards blue/violet colouration in males were significantly lower than expected compared with null expectations (Fig. 3). Thus, our results are consistent with the view of carotenoid colours as key signalling traits that often evolve convergently in response to sexual selection in birds^66^.

Overall, our results highlight the dominant role of sexual selection in driving the evolutionary divergence of avian plumage colouration, and reveal the existence of convergent evolutionary trajectories of avian signalling traits under strong sexual selection. Furthermore, our results suggest that signal divergence under sexual selection may be to some extent predictable, with rapid instances of signal divergence biased towards carotenoid-based colouration traits known to function as honest condition dependent signals of individual quality. Further work is required to assess the generality of these patterns, but given the prevalence of convergently-evolved carotenoid signals within and between avian lineages^64,66^, as well as in other animal lineages including fish and reptiles^67,68^, our results provide insight into the evolutionary forces responsible for explaining patterns of diversity and convergence in visual signalling traits across the animal kingdom.

## Methods

### Specimen selection

We collected data on male and female plumage colouration using study skins from the avian skin collection at the Natural History Museum, Tring. We focused on species for which representatives of both sexes were available and, where possible, sampled specimens of three males and three females, selecting mature individuals in breeding plumage with no obvious signs of moult. Using this approach, we were able to sample specimens of both sexes for 372 (65%) of the taxa represented in the Jetz et al. (ref. ^69^) phylogenies (see Supplementary Methods), with a mean of 2.7 male and 2.4 female specimens per species.

### Digital photography and image processing

Plumage colouration was measured using calibrated digital images of specimens. For full details of equipment and approach, see Supplementary Methods. Following image acquisition, all raw (.NEF) images of specimens were linearised and exported as linear TIFF files using DCRAW^70^. Each linearised image was then normalised using information from the five grey standards included in each image to control for variation in lighting conditions, following the approach of ref. ^71^. We then used these images to extract colour measurements for 10 discrete plumages patches on each specimen (crown, nape, mantle, rump, dorsal tail, wing coverts, wing primaries, throat, breast, and belly). Together these patches provide a comprehensive set of measurements capturing variation in overall plumage colouration for each specimen that is comparable across species^72^. To do this, we used a custom IMAGEJ macro to draw a series of polygons onto our images outlining the location of individual plumage patches on each specimen (Fig. S7). We then used the coordinate locations of these polygons to extract mean RGB values for each patch on each specimen in both the UV and visual range.

### Visual modelling

To convert linearised and normalised RGB values into avian colourspace values, we first estimated the spectral sensitivity of our cameras using data on the camera’s response to a range of colour patches (from commercial colour charts, coloured card, and pastels) of known surface reflectance to estimate camera sensor sensitivity functions (see Supplementary Methods). We then used established methods^71^ to generate mapping functions to convert RGB colour values into avian cone-catch values (Supplementary Table 7). We based our analyses on a violet-sensitive (VS) avian visual system because the available evidence indicates that Tyrannida species are unlikely to possess ultraviolet visual sensitivity^73^. Our dataset therefore consisted of four colour measurements (receptor stimulation values) for 10 plumage patches for a total of 1877 specimens covering 372 species.

To demonstrate the validity of our image-based approach for measuring plumage colouration, we tested whether relative receptor stimulation values extracted from our processed digital images (see below) matched those generated by spectrophotometric measurements of patch reflectance. For a sample of specimens (i.e., species) capturing the breadth of Tyrannida colourspace (*n* = 550 patches; see Supplementary Methods for full details), we found that receptor stimulation values derived from our image-based approach were very highly positively correlated with those based on spectrophotometric data in all four photoreceptor classes (Supplementary Fig. 8; Pearson’s *r* = 0.92–0.96, *P* < 0.001 in all cases). Thus, we conclude that our image-based approach is valid and is capable of providing estimates of patch colouration that are highly similar to those generated using spectrophotometric techniques across the full avian visible range.

We averaged patch values within species (separately for each sex) to generate species-level averages in each variable for each patch for each sex. Following previous studies, we represented chromatic variation among patches using a standard avian colourspace model in which raw cone catch values are converted to relative cone stimuli values and projected in a tetrahedron^74^. This tetrahedron—in which the achromatic (luminance) dimension is removed and each vertex represents one of the four cones characterising avian colour vision (i.e., *v*, *s*, *m*, and *l*)—is the sensory equivalent of a morphospace, where similar colours fall in close proximity in the colourspace and disparate colours are far apart^74^. Given that relative cone stimuli are often correlated, following standard approaches^65^ we conducted a principal component (PC) analysis to represent the colourspace in fewer orthogonal variables. We used the first two PCs, which together accounted for 93% of the variation in the original colourspace variables (Supplementary Table 8). For full details, see Supplementary Methods.

### Trait evolution

We estimated rates of plumage colour evolution using the multivariate version of the variable rates model of trait evolution^75,76^ implemented in the software BayesTraits V2.0.2 (http://www.evolution.rdg.ac.uk/). To provide species-level estimates of plumage evolution rate that also incorporates information on the longer-term rate of trait evolution leading to the phenotype of a given species, we developed an approach for apportioning rate-scaled branch lengths among each tip of a phylogenetic tree^77^. Specifically, our approach involves calculating a weighted average of tip-to-root branch rates for each extant lineage, where, analogously to the equal splits approach^77^, the rate associated with each root-ward edge is down-weighted by a factor of ½:

1 $$m_i = \frac{{\mathop {\sum }\nolimits_{j = 1}^{N_i} \,w_jl_j}}{{\mathop {\sum }\nolimits_{j = 1}^{N_i} \,w_j}}$$

and

2 $$w_j = \frac{1}{{2^{j - 1}}}$$

where *m*_*i*_ is the weighted average of tip *i*, *N*_*i*_ is the number of edges between tip *i* and the root of the tree, and *l*_*j*_ is the length of each edge *j*, with *j* = 1 being the terminal edge leading to the species and *j* *=* *N*_*i*_ being the edge nearest the root. Thus, when applied to phylogenies where branch lengths are in units of rate of trait evolution (rather than time), such as those derived from the outputs of the variable rates model, then these tip-specific weighted average values provide a species-level estimate of trait evolutionary rate that also captures the longer-term rate of trait evolution leading to the phenotype of a given species. We refer to this rate-scaled tip metric as TR_ES_ (Trait Rate equal splits) and apply the above formula to mean rate phenograms derived from outputs of the variable rates models described above.

### Statistical analyses

To assess the relationship between male and female plumage rates (TR_ES_ values) across species, we used phylogenetic reduced major axis regression based on a maximum likelihood optimisation of lambda^78^. To assess the effect of predictor variables on plumage TR_ES_ variation across species, following^31^ we used Bayesian phylogenetic mixed models in MCMCglmm^79^ to fit male and female plumage rates as the response variable using six variables (time since divergence, body mass, dichromatism, forest dependency, latitude, and confamilial sympatry) and their interaction with sex as predictors. We used the same approach to test for interaction effects between dichromatism and colouration category, except that here male and female datasets were analysed separately. For details of methods and data sources, including details regarding classification of patches with respect to signal type, see Supplementary Methods. To test for biases in the evolutionary trajectories of plumage colouration, we quantified the rate, extent, and direction of recent (i.e., branches at the tips of the phylogeny) male and female colour divergence for all species in our dataset based on ancestral values for each colour axis calculated using a maximum likelihood approach^78^. We then compared the average rate of divergence associated with differing evolutionary trajectories in colour space to null distributions of rates generated assuming no relationship between rates and direction of colour evolution. Any deviation in the mean observed rate of evolution compared with the null distribution of rates within each discrete divergence trajectory suggests that rates are disproportionately higher or lower on average than expected relative to null expectations. For full details of this approach, see Supplementary Methods.

## Supplementary information

Supplementary Information

## Data Availability

Data associated with this manuscript are available via Figshare: 10.6084/m9.figshare.7867949.v1.

## Code availability

Scripts used in this manuscript are available via Figshare: 10.6084/m9.figshare.7867949.v1.
