# Supplementary material for: Sexual selection predicts the rate and direction of colour divergence in a large avian radiation
Source: Nat Commun. 2019 Apr 16;10:1773. doi: 10.1038/s41467-019-09859-7 (PMC6467902; doi:10.1038/s41467-019-09859-7)
Supplement: Supplementary file 1 — Supplementary Information [file 41467_2019_9859_MOESM1_ESM.pdf]

SUPPLEMENTARY INFORMATION

Sexual selection predicts the rate and direction of colour divergence in a large avian radiation

Christopher R. Cooney*, Zoë K. Varley, Lara O. Nouri, Christopher J. A. Moody, Michael D. Jardine & Gavin H. Thomas

***Author for correspondence:** c.cooney@sheffield.ac.uk

Supplementary Methods

Phylogenetic framework. We base our analyses on the taxonomy and phylogenies of Jetz et al. [1], which currently represent the only available species-level phylogenetic hypothesis for this group (the Tyrannida) estimated in a consistent framework. The Jetz et al. taxonomy recognizes 575 species in the parvorder Tyrannida[2], which are distributed among three major monophyletic groups: cotingas (Cotingidae, 66 spp.), manakins (Pipridae, 51 spp.) and tyrant-flycatchers and their allies (Tyrannidae; 458 species). To provide a phylogenetic framework for these species, we downloaded 1000 trees produced using a combination of genetic and taxonomic information (that is, those containing all 9,993 species) from http://www.birdtree.org, which were then pruned to generate tree distributions for all species in the group. Due to concerns regarding the use of trees containing species placed using taxonomic information[3], we also downloaded an alternative tree distribution from the same source based on trees containing only the subset of species (396; 69%) placed using genetic data. On the basis of these distributions, we then used TreeAnnotator[4] to generate maximum clade credibility (MCC) trees (using target node heights), which form the basis of our analyses.

Digital photography. Plumage colouration was measured using calibrated digital images of specimens. The calibrated photographs used in this study were collected as part of a broader effort to measure avian colouration, and a digital photography approach was favoured over taking spectrometer measurements as it provides a more efficient way to collect colouration data for large numbers of species, and because whole-specimen photographs facilitate the measurement of both pattern and colouration[5,6], though pattern does not form part of this study. The Nikon D7000 digital single-lens reflex camera used to take the photographs was modified (by Advanced Camera Service, Norfolk; http://advancedcameraservices.co.uk/) to allow both human visible and ultraviolet (UV) wavelengths of light to be recorded. This was achieved by removing the UV filter from the camera sensor, and using a Nikon 105mm f/4.5 UV Nikkor lens. Specimens were illuminated using two Bronocolor Pulso G 1600 J lamps (with UV filters removed) connected to a single Broncolor Scoro 1600 S Power Pack. Two Baader photographic lens filters, permitting human visible (400–680 nm; Baader UV/IR Cut filter / L filter) and UV (320–380 nm; Baader U-Venus-Filter) wavelengths, were used. Each study skin was photographed through both filters from three different angles (dorsal, lateral, ventral), resulting in six images per specimen. The same camera settings were used for all photographs (1/250 sec, f/16.0, ISO 100, 'Daylight' white balance, RAW photo format), with the exception that a higher ISO sensitivity (2000) was used for UV images to achieve correct exposure. Each image contained 5 Labsphere Spectralon Diffuse Reflectance standards of known relative reflectance (2%, 40%, 60%, 80% and 99%) (Supplementary Figure 7), which were used to normalise images by comparing expected pixel intensity values for the grey standards against measured values using regression (forced through the origin).

Camera spectral sensitivity. In order to convert linearised RGB values into avian colourspace values, we first estimated the spectral sensitivity of our cameras. To do this, we followed the method of Pike [7], which uses data on the camera's response to a limited number of colour patches of known surface reflectance to estimate camera sensor sensitivity functions. Three inputs are required: (i) camera response values to multiple colour standards, (ii) spectrophotometric measurements of those same standards, and (iii) the irradiance spectrum of the illuminating light source. To generate camera response values, we first recorded camera responses under normal conditions (see above) to two commercially available colour charts (X-Rite ColourChecker 'Classic' and 'Passport'; X-Rite, Inc.), which together provided 74 individual colour patches. However, as commercial colour charts typically have low reflectivity in the UV (300-400nm) range[7], we also recorded camera responses to two additional setups consisting of pastels and patches of coloured card that possess far greater reflectivity and variability in the UV range[6]. Together these custom-made colour charts consisted of 98 colour patches, giving 172 colour patches in total. Camera response values for each of the color patches were averaged over 20 calibrated images taken in each range (i.e. UV and Vis), post-processed as above. Corresponding reflectance spectra for each colour patch were measured using a USB4000 spectrophotometer, a PX-2 pulsed Xenon light source and a Spectralon white standard (Ocean Optics), and spectra were averaged over 50 measurements with a 20nm boxcar smoothing window applied. Relative irradiance measurements of the flash units were measured using the same setup, after calibrating the device using the output from an iridescent lamp of known colour temperature. Finally, we used these data to estimate camera sensor sensitivity functions (Supplementary Figure 9) for the R, G, and B sensors under both UV and Vis conditions using MATLAB scripts modified from Pike [7] and Martin and Mendelson [8].

Visual modelling. We used methods developed by Troscianko and Stevens [6] to generate mapping functions to convert RGB colour values into avian cone-catch values. This approach works by first estimating camera responses and visual system cone-catch values to a library of natural spectra under a specific illuminate, and then uses multiple regression to create mapping functions for each receptor channel from the camera's responses. Using tools available in the IMAGEJ Image Calibration and Analysis Toolbox (version 1.22)[6], we generated mapping functions for each photoreceptor using equations containing second-order polynomial terms and three-way interactions between channels[6] as these gave the best results when relative cone catch quanta were compared with estimates based on spectrophotometry data (see below). Note that this approach does not incorporate information on camera responses in the UV G channel due to typically low sensitivities of the G channel in the UV range[6]. We fit these equations to our data incorporating information on the estimated spectral sensitivities of our camera and the irradiance spectrum of our illuminant (i.e. flash units). For modelling receptor responses, we assumed idealized illumination conditions[9] and receptor

sensitivities corresponding to an ‘average’ violet-sensitive (VS) avian visual system extracted from the R package *pavo*[10]. The decision to base our analyses on a VS avian visual system was taken because the available genetic and physiological evidence indicates that species in Tyrannida are unlikely to possess ultraviolet visual sensitivity[11,12]. We used this information to generate a mapping function for each cone class, and the resulting models were all characterised by a high degree of mapping accuracy (R^2 values > 0.99; Supplementary Table 7). These mapping functions were then used to convert linearised and normalised RGB values for each patch on each specimen into avian relative receptor stimulation values (v, s, m, l).

Comparison with spectrophotometer-based measurements. We tested whether receptor stimulation values based on our digital image-based approach matched those produced using spectrophotometer measurements of patch reflectance. To do this we used the spectrophotometer setup described above to record patch reflectance spectra for a sample of the same specimens included in this analysis that together captured the breadth of Tyrannida colourspace. We sampled 55 specimens and measured the same 10 plumage patches for each specimen as measured on the images. We took 5 replicate measurements per patch, which we then averaged to give a single mean spectrum per patch. The total dataset therefore consisted of measurements for 550 individual plumage patches (i.e. 55 specimens, 10 patches per specimen). Using functions available in *pavo*[10], spectra were then converted to receptor stimulation values following an analogous pipeline to that used on the image-based data (see above).

Comparing scores for the sample of patches measured using both approaches showed that receptor stimulation values derived from our image-based approach were very strongly correlated with those derived from spec measurements of patch reflectance across all four photoreceptor classes (Supplementary Figure 8). Indeed, the strength of these relationships (Pearson’s r = 0.92-0.96, P < 0.001 in all cases) indicates that our image-based approach is capable of providing estimates of patch colouration that are highly similar to those generated using spectrophotometric techniques across the full avian visible range. We note however that the true correlations between spec- and image-based measurements are likely to be even stronger than these results imply, as the relationships reported here undoubtedly contain an element of noise introduced by slight differences in the precise areas of plumage measured from the images versus the using the spectrophotometer (the latter being capable of providing only point estimates of reflectance).

Models of trait evolution. We estimated rates of plumage colour evolution using the multivariate version of the variable rates model of trait evolution[13,14] implemented in the software BayesTraits V2.0.2 (http://www.evolution.rdg.ac.uk/). This model uses a Bayesian reversible-jump framework to fit a model of trait evolution that allows rates to vary in individual branches or entire monophyletic subgroups of a phylogeny. It has been shown to outperform a variety of other single and multi-process

models of trait evolution when tested on simulated datasets[15], and is frequently observed as the best fitting model in empirical studies[13,14]. Furthermore, variable rates model has the additional benefit that it is capable of generating branch-specific estimates of evolutionary rate for all branches in a tree. We used this approach to estimate rates in two ways. First we estimated overall rates of plumage colour evolution by combining data from all 10 patches into a single analysis. Second we broke our dataset down into constituent plumage patches and fit separate models for each of the 10 body regions individually. Models were fit using default priors and a single-chain Markov chain Monte Carlo (MCMC) run for 1 billion iterations with the first 500 million iterations removed as burnin, allowing correlations between trait axes. From each chain, we sampled parameters every 100,000 iterations and final parameter estimates for each model were based on 5,000 post-burnin samples. We summarised the results of each run by calculating the mean rate for each node in the tree over all posterior samples[14].

Predictor variables. To explore the role of factors predicted to influence colour evolution in birds, we collected data for six key variables. (i) *Time since divergence.* Divergence time estimates for the lineages included in our analysis were generated by extracting the age of the node connecting the species to its sister lineage in the pruned phylogeny. Values were then $\log_{10}$-transformed to improve normality. (ii) *Body mass.* Species-specific estimates of ($\log_{10}$-transformed) body mass were extracted[16]. (iii) S*exual dichromatism.* To quantify the degree of sexual dichromatism within species, we followed previous studies[17-19] and used the summed Euclidean distances between male and female values for each patch. A dichromatism score of zero indicates identical colouration in both sexes (monochromatism) with higher positive values indicating greater degree of dichromatism. To improve normality, dichromatism scores were $\log_{10}$-transformed. (iv) *Forest dependency.* Data on species' forest dependency were compiled from www.datazone.birdlife.org (accessed 18-10-2017). The BirdLife dataset categorizes species into one of four categories: 'low', 'medium' or 'high' forest dependency, or 'does not normally occur in forests'. We converted these data into a binary variable by reclassifying lineages as either non-forest (no or low forest dependency) or forest' (medium or high forest dependency) dependent species. Two species (*Corapipo altera* and *Phaeomyias tumbezana*) were missing data from this source, so for these species we filled the gaps using descriptions in *Handbook of the Birds of the World*[20]. (v) *Latitudinal midpoint.* All variables relating to species' geographical distributions are based on (breeding) range maps provided by BirdLife International and NatureServe (2016, version 9; www.datazone.birdlife.org). We estimated species' latitudinal midpoint[21-23] by calculating range centroids using the R package PBSmapping[24]. (vi) *Confamilial sympatry.* To quantify the extent of interactions with related species, we used the taxonomy of Jetz, et al. [1] and the range maps to tally up the number of same-family (confamilial) species that are sympatric with the focal species[25], where sympatry is defined as >20% range overlap between species—a threshold commonly used to define 'substantial' sympatry[26]. We then divided this number

by the total number of species in the family to provide a species-level metric for the degree of sympatry with related species.

In addition to our six main predictors, we collected data for several other variables chosen to assess the robustness of conclusions, particularly those relating to the role of sexual selection. First, where possible we calculated estimates of intraspecific plumage colour variability using the mean Euclidean distances between same-sex individuals of a given species in terms of cone catch values for each patch, which we used to assess the sensitivity of our main results to the potentially confounding effect of intraspecific variation (or measurement error)[27]. Second, as the approach of summing dichromatism scores (i.e. Euclidean distances) across body regions does not differentiate between cases of moderate dichromatism across many patches versus extensive dichromatism in one or a few body regions, we generated an alternative measure of dichromatism that focuses on the maximum extent of dichromatism across all patches. Third, to test whether similar patterns are recovered using an alternative (i.e. non-colouration based) metric of sexual selection intensity, we extracted data from a recently published study [Dale et al. (2015); ref. [28]] that compiled data on social mating system, sexual size dimorphism and paternal care for thousands of passerine species to generate a composite measure of sexual selection intensity. From this study, data were available for a subset of 130 (35%) species in our dataset.

Categorising patch colouration. For our analyses examining the consistency of the dichromatism-rate relationship among signal types, we assigned each patch in our dataset to one of three non-overlapping colouration categories, with the aim of broadly distinguishing colours consistent with two major colouration mechanisms: (i) carotenoid-based colouration and (ii) structural colouration. To do this, we focused on variation in PC1 scores, as this variable captured the extent to which patch colours primarily stimulated the *l* (red) receptor (negative PC1 scores) versus the *s* and *v* (blue and violet) receptors (positive PC1 scores) (see Supplementary Table 8). In general, mechanistic studies have shown that patches with carotenoid-based colouration have higher values of stimulation of *l* (long wave sensitive) receptor, whereas structurally-coloured patches have higher stimulation of the *s* (short wave sensitive) and *v* (violet sensitive) receptors[9,29]. Accordingly, we defined ‘carotenoid-consistent colours’ [following the terminology of ref. [30]] as any patch falling into the bottom 25% quartile of PC1, which are characterised by high values of *l* receptor stimulation and low values of *v* (or *s*) receptor stimulation (Supplementary Figure 5). Conversely, we defined colours consistent with structural colouration as any patch falling into the top 25% quartile of PC1, which in contrast are characterised by low values of *l* receptor stimulation and high values of *v* (or *s*) receptor stimulation (Supplementary Figure 5). Between these two extremes, we also defined an intermediate colouration category, which is characterised by receptor stimulation values intermediate between those of the other two categories (Supplementary Figure 5). Ideally, we would characterise signal types within our dataset on the basis of detailed knowledge of colour producing mechanisms for the species in our

dataset. However, detailed information regarding colour producing mechanisms (and particularly how such production mechanisms vary among body regions) is lacking for many, if not most, bird species. Thus, inference methods based on the signal properties (i.e. colouration) of plumage patches, such as the one we employ here, are commonly used [e.g. refs. [30,31]] as they currently represent the only feasible way to infer information regarding underlying colour-producing mechanism for large numbers (>100s) of bird species.

Trajectories of colour divergence. To test for biases in the evolution of plumage colouration, we estimated the rate, extent, and direction of recent male and female colour divergence for all species in our dataset. To do this, we estimated ancestral values for each colour axis using a maximum likelihood approach implemented in the R package phytools[32] applied to mean rate-scaled trees produced by fitting the variable rates model. Specifically, following ref. [14], we estimated ancestral states for each species using a Brownian motion model applied to the relevant mean rate-scaled tree for each patch. Ancestral state estimates were based on rate-scaled trees to account for unequal rates of evolution across the tree and among patches, thereby improving estimates of ancestral values. We then used these ancestral values to plot the extent and direction of colour evolution associated with each patch. In this context, the direction of evolution is represented as the angle of bearing of the contemporary value relative to the ancestral value in our two-dimensional colourspace. Alongside this, we also extracted estimates for the evolutionary rate associated with each patch directly from the appropriate mean rate-scaled tree. Throughout this analysis we focused exclusively on evolution at the tips of the phylogeny (i.e. along terminal branches leading to contemporary species) to minimize the problems associated with ancestral trait values among deeper nodes in the tree[33].

Statistical analyses. To assess the relationship between male and female plumage TR_{ES} values across species, we used phylogenetic reduced major axis (RMA) regression based on a maximum likelihood optimization of lambda implemented in phytools[32]. To assess the effect of predictor variables on plumage TR_{ES} variation across species, we used BPMMs in MCMCglmm[34]. Following previous analyses[28], we fit male and female plumage rates as the response variable using the seven variables (sex, time since divergence, body mass, dichromatism, forest dependency, latitude, and confamilial sympatry) as continuous predictors. Phylogenetic effects were considered a random effect, and sex and forest dependency were fit as dummy variables. We ran full models estimating the main effects of all predictors and the interaction effects of sex with all other predictors. Models were fit using flat priors with low levels of belief and were run for 220,000 iterations, sampled every 20 iterations, and with the first 20,000 iterations removed as burnin. Model outputs were checked for convergence and adequate sampling of model parameters. To avoid issues related to model simplification, we limited our analysis to investigation of full models only, and to improve the

interpretability of effects sizes and regression coefficients, predictor variables were centered and standardised prior to model fitting[35,36]. In the main text we present results using the tree topology based on genetic and taxonomic information (see above), but results were qualitatively similar using rates of plumage evolution estimated using a tree topology generated using only genetic data, when accounting for the correlation between intraspecific variation (or measurement error) and rates of plumage colour evolution across species, and when using alternative indices of sexual selection (Supplementary Tables 1-4).

To test the hypothesis that rapid rates of plumage colour evolution are disproportionately associated with divergence in particular directions, we compared the average rate of divergence associated with differing evolutionary trajectories in colour space to null distributions of rates generated assuming no relationship between rates and direction of colour evolution. To do this, we divided up our dataset into discrete 20° bins that together captured the full range of possible divergence trajectories within our two-dimensional colour space. On the basis of these assignments, we then calculated the average rate of evolution associated with each divergence trajectory. To generate null distributions of rates associated with each trajectory, we generated 10,000 replicate datasets in which we randomised the association between divergence direction and rate across species and patches, each time calculating the average rate of evolution within each bin. Thus any deviation in the observed rate of evolution compared to the null distribution of rates within each bin suggests that rates are disproportionately higher or lower on average than expected relative to null expectations.

Supplementary Figures

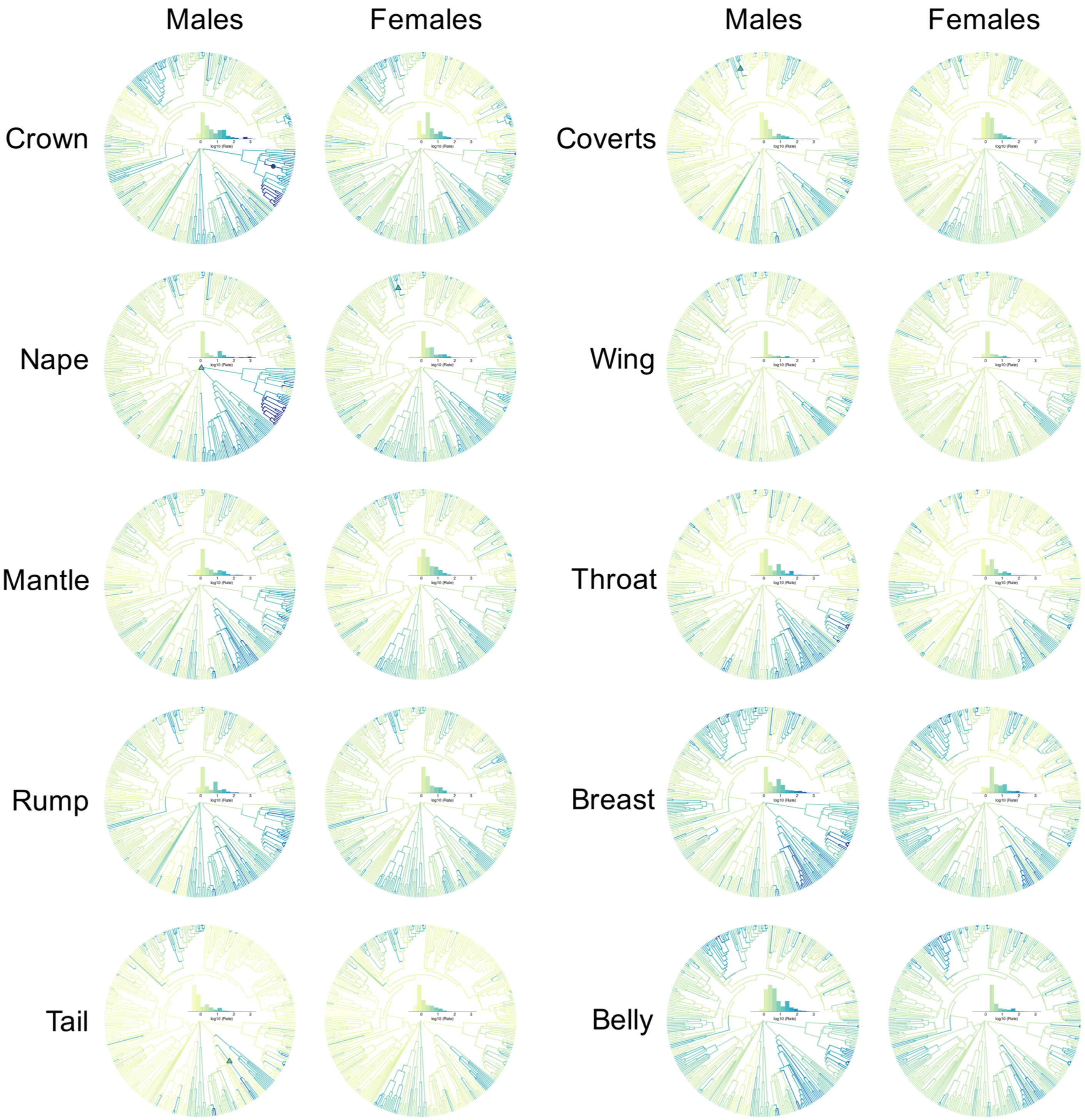

Supplementary Figure 1. Rates of plumage colour evolution within body regions across Tyrannida species. Plots showing the Tyrannida phylogeny (n = 372 spp.) coloured by estimates of mean relative multivariate rate of plumage colour evolution for individual body regions for males and females separately. Coloured shapes show the location of significant (PP > 0.99) rate shifts affecting whole clades (triangles) and individual internal branches (circles) (shape colour indicates rate estimates). Histograms (inset) show the rate distribution for each tree (on a universal scale).

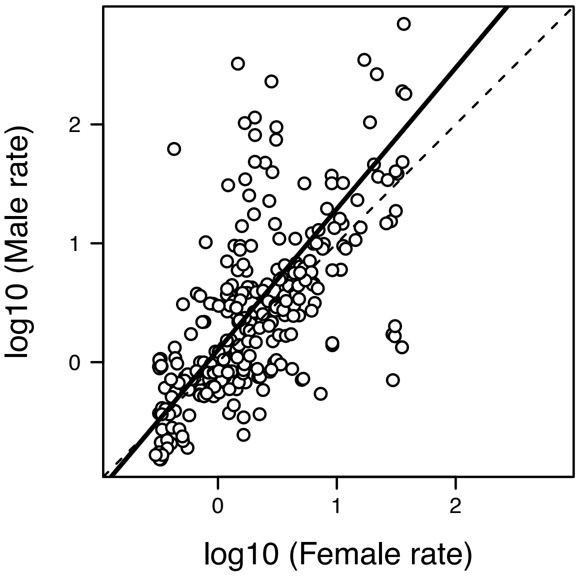

Supplementary Figure 2. The relationship between ($\log_{10}$ transformed) male and female TR_{ES} values of plumage colour evolution across Tyrannida species based on a genetic-only tree topology (n = 290 spp.). Phylogenetic reduced major axis regression (solid) and one-to-one (dashed) lines shown. Equation of the regression line: y = 0.10 + 1.19*x (r^2 = 0.34), which is significantly different to a one-to-one slope (T = 3.57, d.f. = 248.54, P = <0.001).

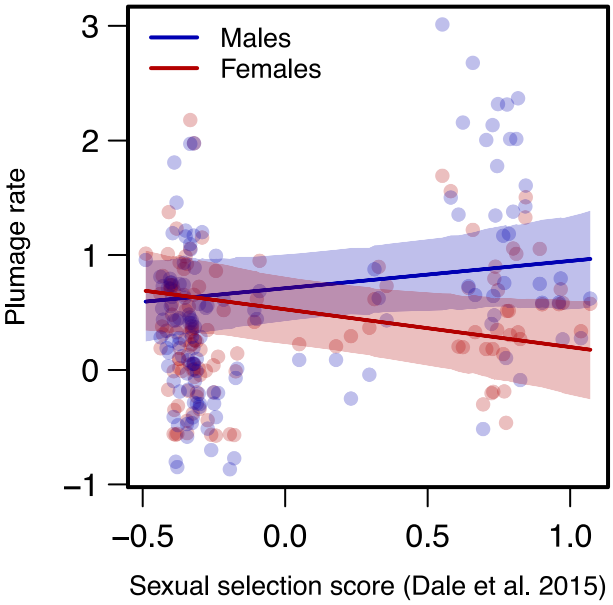

Supplementary Figure 3. The relationship between sexual selection scores from Dale et al. (2015) and rates of plumage colour evolution (n = 130 spp.). Points and regression lines (with 95% credible intervals) are plotted for males (blue) and females (red) separately.

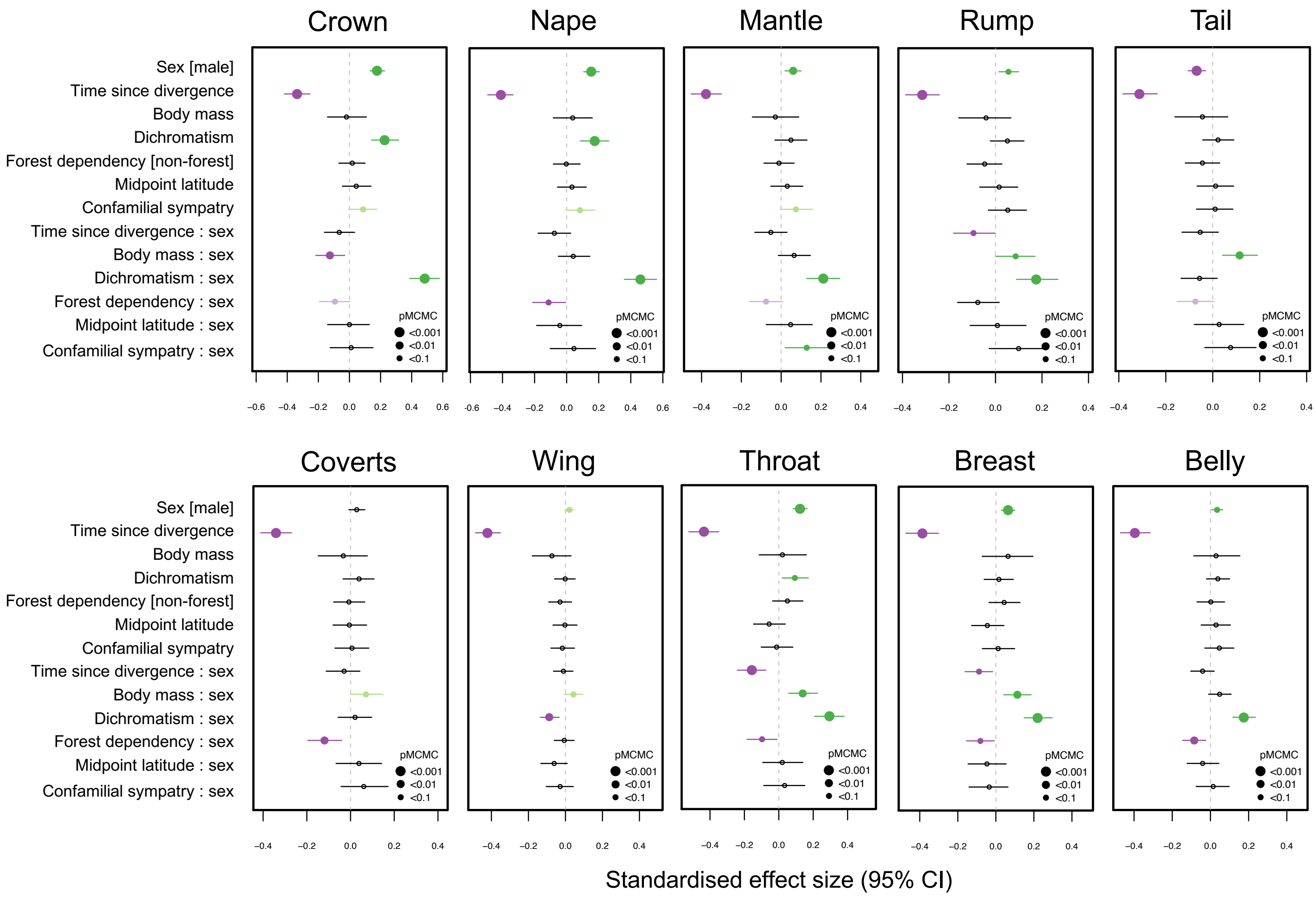

Supplementary Figure 4. Coefficient estimates from multipredictor BPMMs predicting variation in rates of colour evolution (TR_{ES} values) within plumage regions among Tyrannida species (n = 372). Points indicate the mean standardised effect sizes for each of the (scaled) predictor variables and lines indicate 95% credible intervals (CI). Predictors with significant (*pMCMC* < 0.05) effects are coloured purple (negative effect) and green (positive effect).

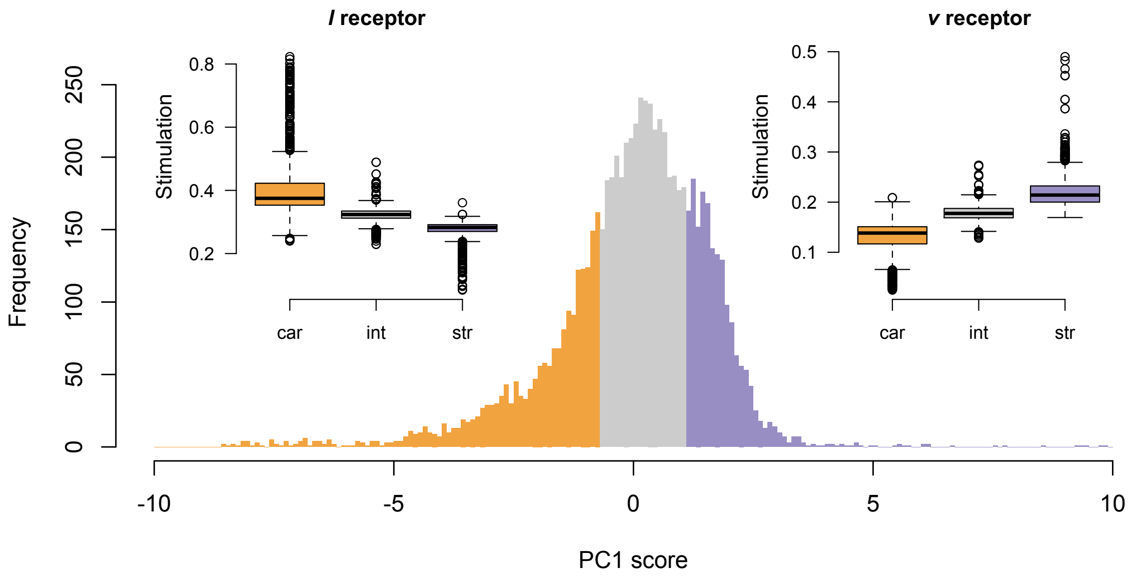

Supplementary Figure 5. A histogram showing the distribution of PC1 scores across plumage patches (n = 7420) for Tyrannida species (n = 372). Bar colours indicate the thresholds used to categorise patches as having carotenoid-consistent colouration (bottom 25% quartile; orange), structural-consistent colouration (top 25% quartile; lilac), or colouration intermediate between these two extremes (middle 50% quartiles; grey). Inset boxplots indicate that patches assigned to the carotenoid-consistent colouration category ("car") are characterised by higher *l* (long wave sensitive) receptor stimulation values associated with red/orange/yellow colouration (left inset), whereas patches assigned to the structural-consistent colouration category (”str”) have higher *v* (violet sensitive) receptor stimulation values associated with blue/violet colouration (right inset). Correspondingly, patches assigned to the intermediate colouration category ("int') have *l* and *v* receptor stimulation values intermediate between these two extremes.

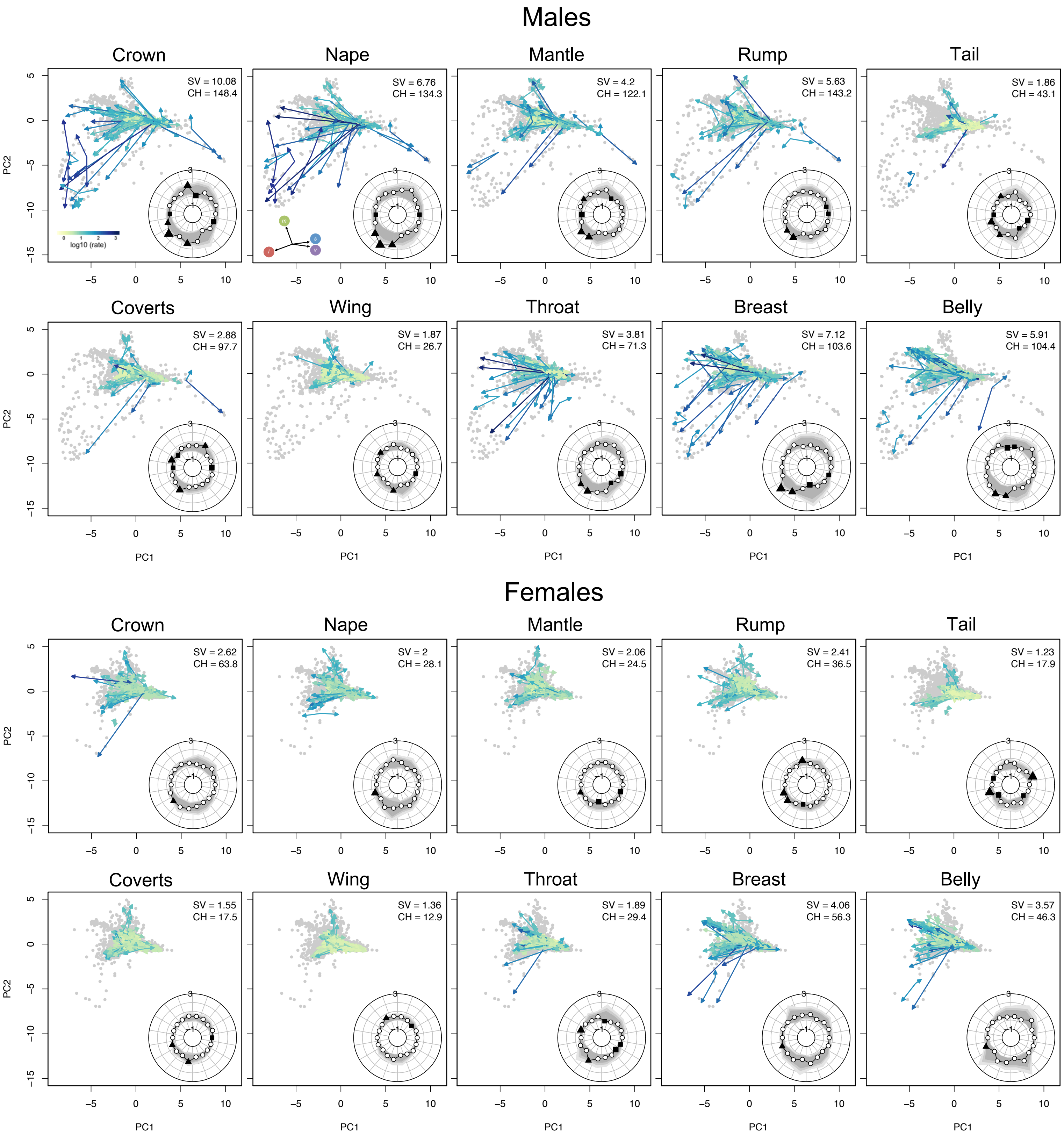

Supplementary Figure 6. Plots showing the rate and trajectory of plumage divergence in colourspace for each sampled plumage patch for males (top) and females (bottom) of 372 Tyrannida species. Arrows indicate the direction of evolution and are coloured according to the corresponding rate of evolution. Grey dots indicate the overall distribution of plumage patch measurements within each sex (for illustrative purposes only). Radar plots (inset) show the mean evolutionary rates of divergence trajectories (black points and lines) falling within each 20° segment of 2-dimensional colourspace. Grey bands indicate confidence intervals for the null distribution of mean rates based on a randomisation process. Filled points indicate values showing significant ($P < 0.05$) deviations from the null distribution, with triangles and squares indicating faster and slower average rates than expected, respectively. Scale bar and PC vectors (inset) are relevant to all panels. SV = sum of variance; CH = convex hull volume.

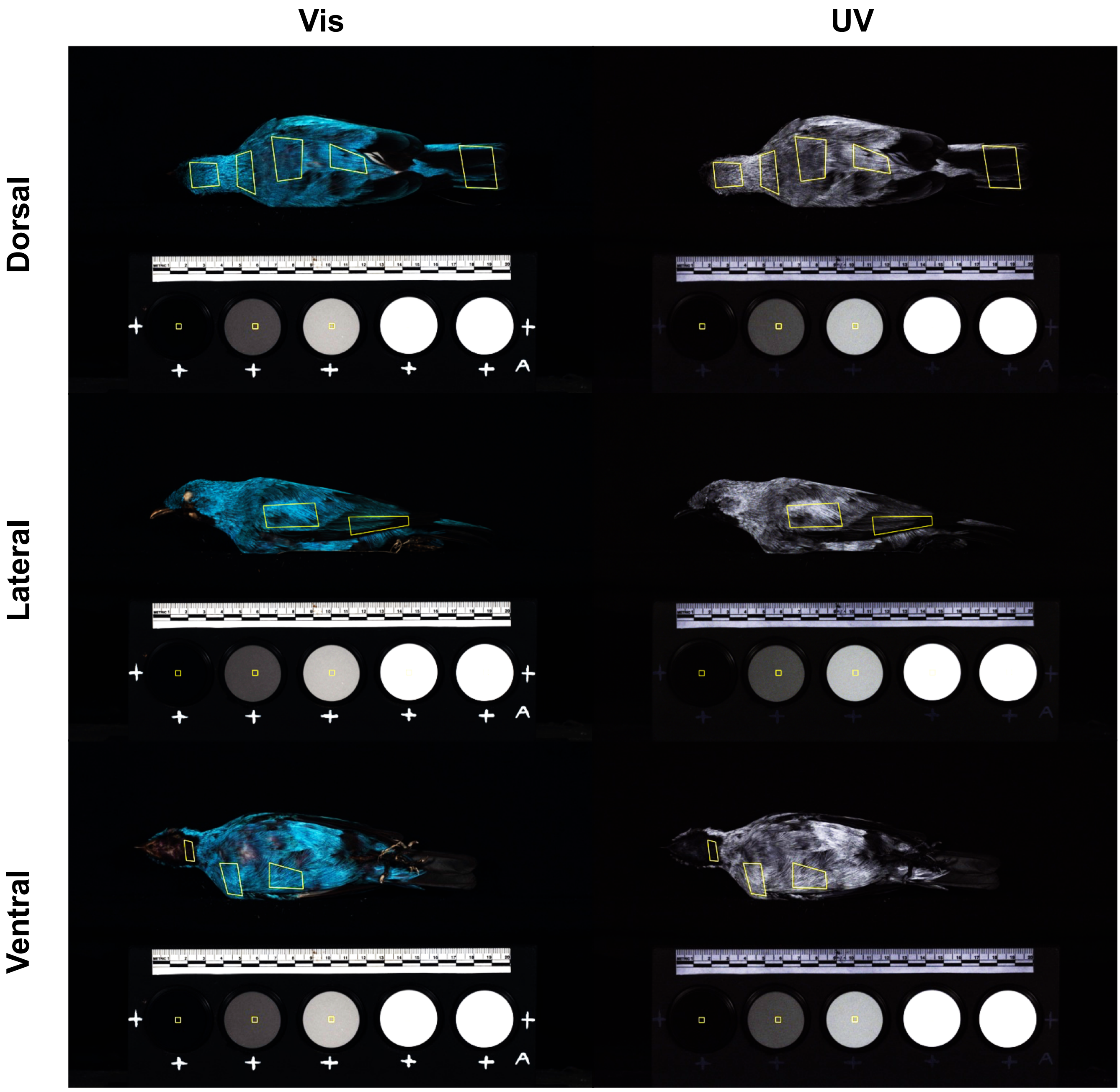

Supplementary Figure 7. Example images showing the patch selection process. We selected 10 patches in total across dorsal (crown, nape, mantle, rump, tail), lateral (coverts, wing) and ventral (throat, breast, belly) views of each specimen, along with the centre point of each of the five grey standards.

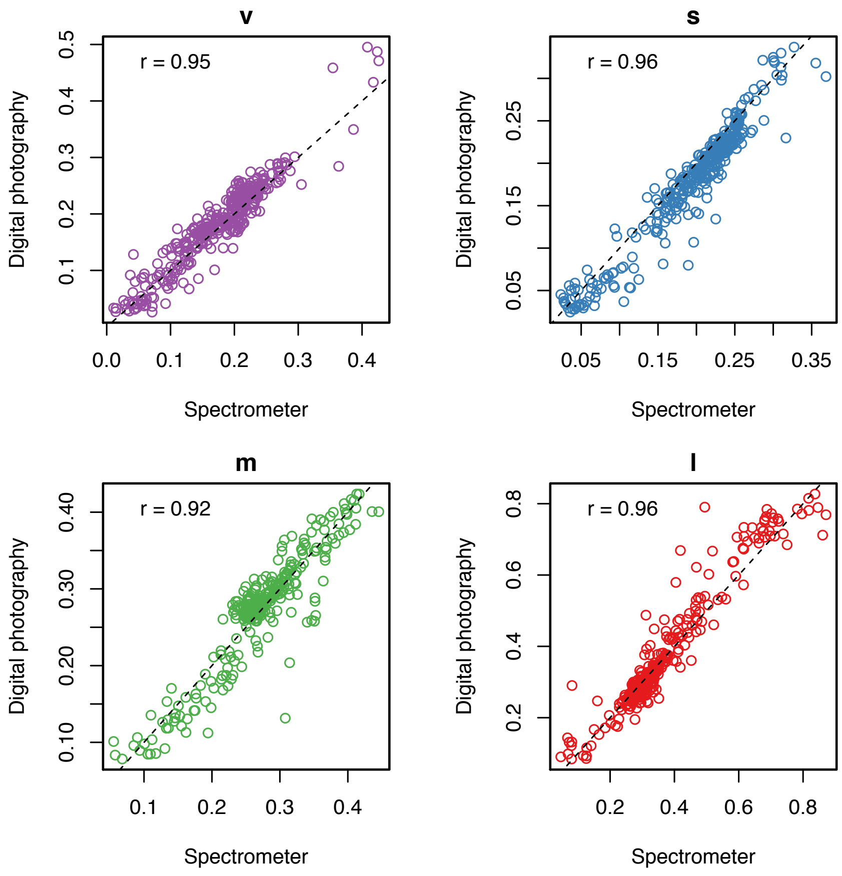

Supplementary Figure 8. The relationship between receptor stimulation values derived from spectrophotometer and digital photography-based reflectance measurements. Points represent data from 550 plumage patches covering 55 Tyrannida species. Inset values show the Pearson correlation coefficient (r) associated with each relationship. Dotted lines indicates a 1:1 relationship.

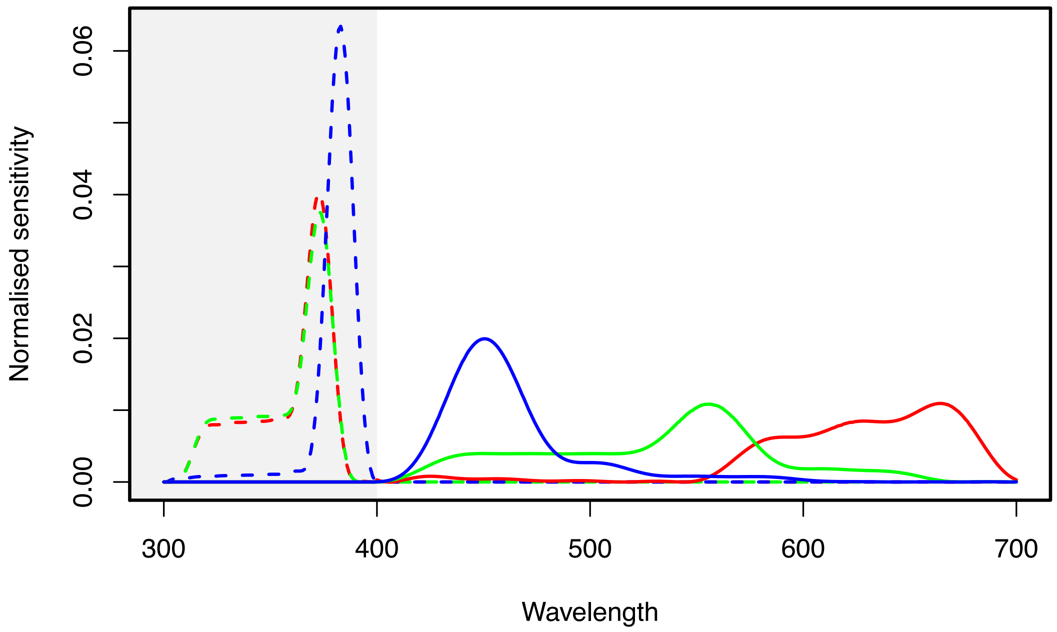

Supplementary Figure 9. Camera sensitivity curves. Plot shows the normalised sensitivity curves for each camera sensor (R, red; G, green; B, blue) for the UV (grey shading; dashed lines) and Vis (solid lines) range.

Supplementary Tables

	Genetic + taxonomic tree (372 spp.)		Genetic only tree (290 spp.)	
Term	Estimate (CI)	*pMCMC*	Estimate (CI)	*pMCMC*
(Intercept)	0.59 [0.36, 0.85]	**<0.001*****	0.50 [0.23, 0.78]	**0.001****
Sex [male]	0.12 [0.06, 0.17]	**<0.001*****	0.10 [0.04, 0.17]	**0.002****
Time since divergence	-0.52 [-0.62, -0.42]	**<0.001*****	-0.40 [-0.52, -0.28]	**<0.001*****
Body mass	-0.07 [-0.22, 0.08]	0.366	0.08 [-0.10, 0.26]	0.373
Dichromatism	0.33 [0.20, 0.46]	**<0.001*****	0.25 [0.11, 0.39]	**<0.001*****
Forest dependency [non-forest]	0.03 [-0.08, 0.13]	0.628	0.04 [-0.08, 0.15]	0.510
Midpoint latitude	0.11 [0.00, 0.22]	**0.046***	-0.04 [-0.19, 0.09]	0.546
Confamilial sympatry	0.14 [0.03, 0.25]	**0.012***	0.05 [-0.10, 0.19]	0.493
Time since divergence : sex	-0.05 [-0.17, 0.06]	0.405	-0.06 [-0.20, 0.07]	0.369
Body mass : sex	-0.04 [-0.16, 0.07]	0.507	0.05 [-0.08, 0.17]	0.481
Dichromatism : sex	0.41 [0.29, 0.53]	**<0.001*****	0.35 [0.21, 0.48]	**<0.001*****
Forest dependency : sex	-0.06 [-0.18, 0.05]	0.306	-0.09 [-0.23, 0.04]	0.181
Midpoint latitude : sex	0.02 [-0.15, 0.18]	0.846	-0.07 [-0.27, 0.13]	0.518
Confamilial sympatry : sex	0.01 [-0.15, 0.18]	0.899	-0.09 [-0.30, 0.12]	0.392

Supplementary Table 1. Standardised coefficient estimates from a Bayesian phylogenetic mixed-model (BPMM) predicting variation in evolutionary rates (TR_{ES} values) among Tyrannida species. CI, credible interval. ***, *pMCMC* < 0.001; **, *pMCMC* < 0.01; *, *pMCMC* < 0.05. Marginal R^2 values (i.e. variance explained by fixed factors) for full models are 0.23 and 0.18, respectively.

	Genetic + taxonomic tree (349 spp.)		Genetic only tree (276 spp.)	
Term	Estimate (CI)	*pMCMC*	Estimate (CI)	*pMCMC*
(Intercept)	0.53 [0.29, 0.75]	**<0.001***	0.46 [0.21, 0.71]	**<0.001***
Sex [male]	0.12 [0.06, 0.18]	**<0.001***	0.13 [0.06, 0.19]	**<0.001***
Time since divergence	-0.51 [-0.60, -0.41]	**<0.001***	-0.44 [-0.55, -0.33]	**<0.001***
Body mass	0.00 [-0.17, 0.17]	0.990	0.11 [-0.06, 0.29]	0.201
Dichromatism	0.16 [0.03, 0.30]	**0.015***	0.09 [-0.07, 0.24]	0.263
Forest dependency [non-forest]	0.07 [-0.04, 0.17]	0.220	0.07 [-0.05, 0.18]	0.261
Midpoint latitude	0.06 [-0.06, 0.17]	0.342	-0.08 [-0.23, 0.06]	0.264
Confamilial sympatry	0.08 [-0.03, 0.20]	0.159	-0.03 [-0.18, 0.12]	0.674
Intraspecific variation	0.33 [0.25, 0.41]	**<0.001***	0.34 [0.25, 0.43]	**<0.001***
Time since divergence : sex	-0.05 [-0.18, 0.07]	0.433	-0.09 [-0.22, 0.06]	0.210
Body mass : sex	-0.02 [-0.14, 0.10]	0.704	0.08 [-0.05, 0.22]	0.238
Dichromatism : sex	0.43 [0.30, 0.55]	**<0.001***	0.35 [0.22, 0.50]	**<0.001***
Forest dependency : sex	-0.05 [-0.18, 0.08]	0.384	-0.10 [-0.23, 0.05]	0.178
Midpoint latitude : sex	0.06 [-0.11, 0.24]	0.512	-0.06 [-0.27, 0.18]	0.625
Confamilial sympatry : sex	-0.02 [-0.20, 0.15]	0.849	-0.10 [-0.32, 0.13]	0.389

Supplementary Table 2. Standardised coefficient estimates from a Bayesian phylogenetic mixed-model (BPMM) predicting variation in evolutionary rates (TR_{ES} values) among Tyrannida species for which estimates of (sex-specific) intraspecific variation were available. CI, credible interval. ***, *pMCMC* < 0.001; **, *pMCMC* < 0.01; *, *pMCMC* < 0.05. Marginal R^2 values (i.e. variance explained by fixed factors) for full models are 0.27 and 0.24, respectively.

	Genetic + taxonomic tree (372 spp.)		Genetic only tree (290 spp.)	
Term	Estimate (CI)	*pMCMC*	Estimate (CI)	*pMCMC*
(Intercept)	0.57 [0.32, 0.79]	**<0.001***	0.48 [0.21, 0.73]	**0.001**
Sex [male]	0.12 [0.06, 0.17]	**<0.001***	0.11 [0.04, 0.17]	**0.001**
Time since divergence	-0.52 [-0.61, -0.42]	**<0.001***	-0.41 [-0.53, -0.30]	**<0.001***
Body mass	-0.04 [-0.19, 0.10]	0.590	0.09 [-0.09, 0.26]	0.309
Maximum dichromatism	0.38 [0.27, 0.50]	**<0.001***	0.35 [0.22, 0.49]	**<0.001***
Forest dependency [non-forest]	0.03 [-0.07, 0.13]	0.595	0.03 [-0.07, 0.14]	0.550
Midpoint latitude	0.14 [0.03, 0.24]	**0.016***	-0.02 [-0.15, 0.12]	0.825
Confamilial sympatry	0.16 [0.04, 0.26]	**0.005***	0.07 [-0.07, 0.21]	0.342
Time since divergence : sex	-0.04 [-0.16, 0.08]	0.477	-0.06 [-0.20, 0.07]	0.371
Body mass : sex	-0.03 [-0.15, 0.08]	0.578	0.04 [-0.09, 0.17]	0.577
Maximum dichromatism : sex	0.43 [0.31, 0.55]	**<0.001***	0.42 [0.29, 0.56]	**<0.001***
Forest dependency : sex	-0.07 [-0.19, 0.04]	0.262	-0.09 [-0.22, 0.04]	0.165
Midpoint latitude : sex	0.04 [-0.13, 0.21]	0.599	-0.03 [-0.23, 0.18]	0.771
Confamilial sympatry : sex	0.02 [-0.15, 0.18]	0.793	-0.08 [-0.29, 0.13]	0.433

Supplementary Table 3. Standardised coefficient estimates from a Bayesian phylogenetic mixed-model (BPMM) predicting variation in evolutionary rates (TR_{ES} values) among Tyrannida species using maximum dichromatism across patches. CI, credible interval. ***, *pMCMC* < 0.001; **, *pMCMC* < 0.01; *, *pMCMC* < 0.05. Marginal R^2 values (i.e. variance explained by fixed factors) for full models are 0.25 and 0.23, respectively.

	Genetic + taxonomic tree (130 spp.)		Genetic only tree (115 spp.)	
Term	Estimate (CI)	*pMCMC*	Estimate (CI)	*pMCMC*
(Intercept)	0.62 [0.34, 0.92]	**<0.001*****	0.51 [0.23, 0.78]	**<0.001*****
Sex [male]	0.18 [0.08, 0.29]	**0.001****	0.14 [0.04, 0.24]	**0.004****
Time since divergence	-0.52 [-0.69, -0.36]	**<0.001*****	-0.43 [-0.58, -0.26]	**<0.001*****
Body mass	0.07 [-0.24, 0.38]	0.637	0.14 [-0.12, 0.39]	0.294
Dale sexual selection score	-0.05 [-0.34, 0.26]	0.776	0.12 [-0.14, 0.38]	0.377
Forest dependency [non-forest]	0.05 [-0.14, 0.22]	0.624	0.13 [-0.02, 0.30]	0.106
Midpoint latitude	-0.24 [-0.49, 0.02]	0.069	-0.51 [-0.76, -0.26]	**<0.001*****
Confamilial sympatry	-0.21 [-0.47, 0.04]	0.095	-0.34 [-0.59, -0.08]	**0.009****
Time since divergence : sex	0.02 [-0.19, 0.24]	0.883	-0.15 [-0.36, 0.06]	0.161
Body mass : sex	-0.13 [-0.35, 0.10]	0.258	0.14 [-0.07, 0.35]	0.189
Sexual selection score : sex	0.57 [0.32, 0.82]	**<0.001*****	0.44 [0.20, 0.67]	**0.001****
Forest dependency : sex	0.09 [-0.13, 0.32]	0.442	0.08 [-0.13, 0.31]	0.473
Midpoint latitude : sex	-0.01 [-0.38, 0.37]	0.967	-0.40 [-0.77, -0.01]	**0.037***
Confamilial sympatry : sex	0.01 [-0.38, 0.38]	0.946	-0.36 [-0.76, 0.02]	0.071

Supplementary Table 4. Standardised coefficient estimates from a Bayesian phylogenetic mixed-model (BPMM) predicting variation in evolutionary rates (TR_{ES} values) among Tyrannida species for which sexual selection scores from Dale et al (2015) were available. CI, credible interval. ***, *pMCMC* < 0.001; **, *pMCMC* < 0.01; *, *pMCMC* < 0.05. Marginal R^2 values (i.e. variance explained by fixed factors) for full models are 0.20 and 0.25, respectively.

Trajectory (bearing; °)	Males			Females		
	Mean rate	Null CI (95%)	*P* (lower, upper)	Mean rate	Null CI (95%)	*P* (lower, upper)
0-20	0.49	0.34, 0.70	0.365, 0.635	0.47	0.33, 0.59	0.581, 0.419
20-40	0.45	0.37, 0.69	0.168, 0.832	0.26	0.32, 0.60	**0.002**, 0.998
40-60	0.48	0.40, 0.67	0.269, 0.732	0.40	0.34, 0.57	0.196, 0.804
60-80	0.45	0.43, 0.62	0.057, 0.943	0.50	0.38, 0.53	0.862, 0.138
80-100	0.40	0.47, 0.58	**<0.001**, 1.000	0.39	0.42, 0.49	**<0.001**, 1.000
100-120	0.46	0.48, 0.57	**0.002**, 0.998	0.43	0.42, 0.49	0.076, 0.924
120-140	0.46	0.43, 0.63	0.104, 0.896	0.34	0.37, 0.54	**0.003**, 0.997
140-160	0.29	0.38, 0.68	**0.001**, 0.999	0.33	0.33, 0.59	**0.028**, 0.972
160-180	0.42	0.33, 0.73	0.150, 0.850	0.40	0.30, 0.60	0.219, 0.781
180-200	0.78	0.36, 0.69	0.998, **0.002**	0.47	0.30, 0.61	0.568, 0.432
200-220	1.07	0.39, 0.66	1.000, **<0.001**	0.63	0.31, 0.60	0.988, **0.012**
220-240	0.98	0.40, 0.66	1.000, **<0.001**	0.47	0.35, 0.57	0.594, 0.406
240-260	0.63	0.45, 0.61	0.995, **0.005**	0.64	0.39, 0.51	1.000, **<0.001**
260-280	0.40	0.47, 0.58	**<0.001**, 1.000	0.39	0.41, 0.50	**0.001**, 0.999
280-300	0.62	0.48, 0.58	1.000, **<0.001**	0.53	0.42, 0.50	1.000, **<0.001**
300-320	0.58	0.45, 0.61	0.922, 0.078	0.50	0.40, 0.52	0.909, 0.091
320-340	0.60	0.42, 0.64	0.911, 0.089	0.48	0.36, 0.56	0.680, 0.320
340-360	0.63	0.37, 0.67	0.920, 0.080	0.57	0.33, 0.58	0.966, **0.035**

Supplementary Table 5. Mean (log_{10} transformed) rate estimates associated with different trajectories of colour evolution. Divergence trajectories were measured by calculating angles of divergence within two-dimensional colourspace (see Fig. 3, main text), based on a tree topology generated using genetic and taxonomic information (n = 372 spp.). Null confidence interval (CI) values refer to the 95% CIs for the null distribution of mean rates based on a randomisation process. *P* values indicate whether the observed mean rate in each trajectory class is significantly lower or higher than expected based on null expectations. Significant *P* values ($P < 0.05$) are highlighted in bold.

	Males			Females		
Trajectory (bearing; °)	Mean rate	Null CI (95%)	*P* (lower, upper)	Mean rate	Null CI (95%)	*P* (lower, upper)
0-20	0.64	0.28, 0.65	0.963, **0.037**	0.40	0.25, 0.58	0.511, 0.489
20-40	0.52	0.27, 0.67	0.709, 0.291	0.31	0.26, 0.55	0.106, 0.894
40-60	0.34	0.30, 0.63	0.050, 0.950	0.26	0.27, 0.53	**0.012**, 0.988
60-80	0.49	0.36, 0.58	0.645, 0.355	0.39	0.33, 0.48	0.424, 0.576
80-100	0.31	0.41, 0.53	**<0.001**, 1.000	0.34	0.36, 0.44	**0.003**, 0.998
100-120	0.39	0.42, 0.52	**0.002**, 0.998	0.39	0.37, 0.44	0.343, 0.657
120-140	0.38	0.36, 0.59	0.058, 0.942	0.24	0.31, 0.50	**<0.001**, 1.000
140-160	0.37	0.30, 0.64	0.137, 0.863	0.25	0.25, 0.55	**0.020**, 0.980
160-180	0.39	0.28, 0.66	0.208, 0.792	0.37	0.24, 0.57	0.352, 0.648
180-200	0.80	0.28, 0.67	0.999, **0.001**	0.23	0.23, 0.59	**0.026**, 0.974
200-220	1.24	0.32, 0.63	1.000, **<0.001**	0.58	0.26, 0.55	0.992, **0.008**
220-240	0.82	0.32, 0.63	1.000, **<0.001**	0.51	0.28, 0.52	0.960, **0.040**
240-260	0.49	0.38, 0.56	0.673, 0.327	0.57	0.34, 0.47	1.000, **<0.001**
260-280	0.35	0.41, 0.53	**<0.001**, 1.000	0.32	0.36, 0.44	**<0.001**, 1.000
280-300	0.56	0.41, 0.52	0.999, **0.001**	0.48	0.36, 0.44	1.000, **<0.001**
300-320	0.51	0.39, 0.56	0.837, 0.163	0.47	0.34, 0.47	0.985, **0.015**
320-340	0.55	0.35, 0.60	0.891, 0.110	0.40	0.30, 0.51	0.484, 0.516
340-360	0.62	0.31, 0.63	0.964, **0.036**	0.60	0.27, 0.53	0.998, **0.002**

Supplementary Table 6. Mean (log_{10} transformed) rate estimates associated with different trajectories of colour evolution for species in the phylogeny represented by genetic data. Divergence trajectories were measured by calculating angles of divergence within two-dimensional colourspace (see Fig. 3, main text), based on a tree topology generated using only genetic information (n = 290 spp.). Null confidence interval (CI) values refer to the 95% CIs for the null distribution of mean rates based on a randomisation process. *P* values indicate whether the observed mean rate in each trajectory class is significantly lower or higher than expected based on null expectations. Significant *P* values ($P < 0.05$) are highlighted in bold.

Receptor	R^2
v	0.99875
s	0.99871
m	0.99989
l	0.99872

Supplementary Table 7. The accuracy of fitted cone mapping models for each avian receptor class. *v*, violet sensitive; *s*, short wave sensitive; *m*, medium wave sensitive; *l*, long wave sensitive.

Variable	PC1	PC2
v	0.57	-0.13
s	0.57	0.07
m	-0.20	0.91
l	-0.55	-0.39
Variance (%)	68.29	26.90

Supplementary Table 8. PCA results. Variable loadings and variance explained by the first two principal components (PCs) of an analysis of relative cone stimulation values for the four avian colour cones (*v*, *s*, *m*, *l*) based on 7440 measurements of avian plumage colouration.

Supplementary References

1 Jetz, W., Thomas, G. H., Joy, J. B., Hartmann, K. & Mooers, A. O. The global diversity of birds in space and time. *Nature* **491**, 444-448, (2012).

2 Ericson, P. G. P. *et al.* Higher-level phylogeny and morphological evolution of tyrant flycatchers, cotingas, manakins, and their allies (Aves: Tyrannida). *Mol. Phylogen. Evol.* **40**, 471-483, (2006).

3 Rabosky, D. L. No substitute for real data: a cautionary note on the use of phylogenies from birth-death polytomy resolvers for downstream comparative analyses. *Evolution* **69**, 3207-3216, (2015).

4 Drummond, A. J., Suchard, M. A., Xie, D. & Rambaut, A. Bayesian phylogenetics with BEAUti and the BEAST 1.7. *Mol. Biol. Evol.* **29**, 1969-1973, (2012).

5 Stevens, M., Párraga, C. A., Cuthill, I. C., Partridge, J. C. & Troscianko, T. S. Using digital photography to study animal coloration. *Biol. J. Linn. Soc.* **90**, 211-237, (2007).

6 Troscianko, J. & Stevens, M. Image calibration and analysis toolbox – a free software suite for objectively measuring reflectance, colour and pattern. *Methods in Ecology and Evolution* **6**, 1320-1331, (2015).

7 Pike, T. W. Using digital cameras to investigate animal colouration: estimating sensor sensitivity functions. *Behav. Ecol. Sociobiol.* **65**, 849-858, (2011).

8 Martin, M. D. & Mendelson, T. C. Changes in sexual signals are greater than changes in ecological traits in a dichromatic group of fishes. *Evolution* **68**, 3618-3628, (2014).

9 Stoddard, M. C. & Prum, R. O. Evolution of avian plumage color in a tetrahedral color space: a phylogenetic analysis of New World buntings. *Am. Nat.* **171**, 755-776, (2008).

10 Maia, R., Eliason, C. M., Bitton, P. P., Doucet, S. M. & Shawkey, M. D. pavo: an R package for the analysis, visualization and organization of spectral data. *Methods in Ecology and Evolution* **4**, 906-913, (2013).

11 Ödeen, A., Håstad, O. & Alström, P. Evolution of ultraviolet vision in the largest avian radiation - the passerines. *BMC Evol. Biol.* **11**, 313, (2011).

12 Borges, R. *et al.* Gene loss, adaptive evolution and the co-evolution of plumage coloration genes with opsins in birds. *BMC Genomics* **16**, 751, (2015).

13 Venditti, C., Meade, A. & Pagel, M. Multiple routes to mammalian diversity. *Nature* **479**, 393-396, (2011).

14 Cooney, C. R. *et al.* Mega-evolutionary dynamics of the adaptive radiation of birds. *Nature* **542**, 344-347, (2017).

15 Chira, A. M. & Thomas, G. H. The impact of rate heterogeneity on inference of phylogenetic models of trait evolution. *J. Evol. Biol.* **29**, 2502-2518, (2016).

16 Wilman, H. *et al.* EltonTraits 1.0: species-level foraging attributes of the world's birds and mammals. *Ecology* **95**, 2027, (2014).

17 Cooney, C. R., Tobias, J. A., Weir, J. T., Botero, C. A. & Seddon, N. Sexual selection, speciation and constraints on geographical range overlap in birds. *Ecol. Lett.* **20**, 863-871, (2017).

18 Seddon, N. *et al.* Sexual selection accelerates signal evolution during speciation in birds. *Proc. R. Soc. London Ser. B* **280**, 20131065, (2013).

19 Huang, H. & Rabosky, D. L. Sexual selection and diversification: reexamining the correlation between dichromatism and speciation rate in birds. *Am. Nat.* **184**, E101-E114, (2014).

20 del Hoyo, J., Elliott, A., Sargatal, J. & Christe, D. A. *The Handbook of the Birds of the World, vols 1-16*. (Lynx Edicions, 1992–2011).

21 Weir, J. T. & Schluter, D. The latitudinal gradient in recent speciation and extinction rates of birds and mammals. *Science* **315**, 1574-1576, (2007).
22 Weir, J. T. & Wheatcroft, D. A latitudinal gradient in rates of evolution of avian syllable diversity and song length. *Proc. R. Soc. London Ser. B* **278**, 1713-1720, (2011).
23 Weir, J. T., Wheatcroft, D. J. & Price, T. D. The role of ecological constraint in driving the evolution of avian song frequency across a latitudinal gradient. *Evolution* **66**, 2773-2783, (2012).
24 Schnute, J. T. PBSmapping: mapping fisheries data and spatial analysis tools. R package version 2.69.76. https://CRAN.R-project.org/package=PBSmapping. (2015).
25 Morinay, J., Cardoso, G. C., Doutrelant, C. & Covas, R. The evolution of birdsong on islands. *Ecology and Evolution* **3**, 5127-5140, (2013).
26 Tobias, J. A. *et al.* Species coexistence and the dynamics of phenotypic evolution in adaptive radiation. *Nature* **506**, 359-363, (2014).
27 Silvestro, D., Kostikova, A., Litsios, G., Pearman, P. B. & Salamin, N. Measurement errors should always be incorporated in phylogenetic comparative analysis. *Methods in Ecology and Evolution* **6**, 340-346, (2015).
28 Dale, J., Dey, C. J., Delhey, K., Kempenaers, B. & Valcu, M. The effects of life history and sexual selection on male and female plumage colouration. *Nature* **527**, 367-370, (2015).
29 Stoddard, M. C. & Prum, R. O. How colorful are birds? Evolution of the avian plumage color gamut. *Behav. Ecol.* **22**, 1042-1052, (2011).
30 Thomas, D. B. *et al.* Ancient origins and multiple appearances of carotenoid-pigmented feathers in birds. *Proc. R. Soc. London Ser. B* **281**, 20140806, (2014).
31 Olson, V. A. & Owens, I. P. Interspecific variation in the use of carotenoid-based coloration in birds: diet, life history and phylogeny. *J. Evol. Biol.* **18**, 1534-1546, (2005).
32 Revell, L. J. phytools: an R package for phylogenetic comparative biology (and other things). *Methods in Ecology and Evolution* **3**, 217-223, (2012).
33 Losos, J. B. Seeing the forest for the trees: the limitations of phylogenies in comparative biology. *Am. Nat.* **177**, 709-727, (2011).
34 Hadfield, J. D. & Nakagawa, S. General quantitative genetic methods for comparative biology: phylogenies, taxonomies and multi-trait models for continuous and categorical characters. *J. Evol. Biol.* **23**, 494-508, (2010).
35 Grueber, C. E., Nakagawa, S., Laws, R. J. & Jamieson, I. G. Multimodel inference in ecology and evolution: challenges and solutions. *J. Evol. Biol.* **24**, 699-711, (2011).
36 Schielzeth, H. Simple means to improve the interpretability of regression coefficients. *Methods in Ecology and Evolution* **1**, 103-113, (2010).
